# Characteristics of hydrate-bound gas retrieved at the Kedr mud volcano (southern Lake Baikal)

**DOI:** 10.1038/s41598-020-71410-2

**Published:** 2020-09-08

**Authors:** Akihiro Hachikubo, Hirotsugu Minami, Satoshi Yamashita, Andrey Khabuev, Alexey Krylov, Gennadiy Kalmychkov, Jeffrey Poort, Marc De Batist, Alexandr Chenskiy, Andrey Manakov, Oleg Khlystov

**Affiliations:** 1 Kitami Institute of Technology, 165 Koen-cho, Kitami, 090-8507 Japan; 2 Limnological Institute, SB RAS, 3 Ulan-Batorskaya St, Irkutsk, Russia 664033; 3 Institute of Earth Sciences, St. Petersburg State University, 7-9, Universitetskaya Nab., St. Petersburg, Russia 199034; 4 VNIIOkeangeologia, Anglyisky prospect 1, St. Petersburg, Russia 190121; 5 Vinogradov Institute of Geochemistry, SB RAS, 1-a Favorsky St, Irkutsk, Russia 664033; 6 Sorbonne Université, CNRS, Institut des Sciences de la Terre de Paris, ISTeP, 4 place Jussieu, 75005 Paris, France; 7 Renard Centre of Marine Geology, Ghent University, Krijgslaan 281 s8, 9000 Ghent, Belgium; 8 Irkutsk National Research Technical University, 83 Lemontov St, Irkutsk, Russia 664074; 9 Nikolaev Institute of Inorganic Chemistry, SB RAS, 3 Acad. Lavrentiev Ave, Novosibirsk, Russia 630090

**Keywords:** Biogeochemistry, Environmental sciences, Energy science and technology

## Abstract

We reported the characteristics of hydrate-bound hydrocarbons in lake-bottom sediments at the Kedr mud volcano in Lake Baikal. Twenty hydrate-bearing sediment cores were retrieved, and methane-stable isotopes of hydrate-bound gases (δ^13^C and δ^2^H of − 47.8‰ to − 44.0‰ V-PDB and − 280.5‰ to − 272.8‰ V-SMOW, respectively) indicated their thermogenic origin accompanied with secondary microbial methane. Powder X-ray diffraction patterns of the crystals and molecular composition of the hydrate-bound gases suggested that structure II crystals showed a high concentration of ethane (around 14% of hydrate-bound hydrocarbons), whereas structure I crystals showed a relatively low concentration of ethane (2–5% of hydrate-bound hydrocarbons). These different crystallographic structures comprised complicated layers in the sub-lacustrine sediment, suggesting that the gas hydrates partly dissociate, concentrate ethane and form structure II crystals. We concluded that a high concentration of thermogenic ethane primarily controls the crystallographic structure of gas hydrates and that propane, iso-butane (2-methylpropane) and neopentane (2,2-dimethylpropane) are encaged into crystals in the re-crystallisation process.

## Introduction

Gas hydrates are crystalline clathrate compounds composed of water and gas molecules that are stable at low temperature and high partial pressure of each gas component^1^. Natural gas hydrates, which contain methane (C_1_) as a major component, exist in sea/lake sediment columns and permafrost layers and are considered to be a possible global source of energy^2,3^. There are different views on the role of gas hydrates on global warming^4–6^. They are of concern as a large reservoir of C_1_; however, the amount of hydrate-bound gas is smaller than that expected previously, which makes them unlikely to cause global warming by dissociation of C_1_^5,6^. Moreover, the current understanding of the formation, dissociation and maintenance processes of natural gas hydrates is still incomplete.

Molecular fractionation during formation of gas hydrate crystals occurs according to the size ratio of guest molecules to host cages and the difference in equilibrium pressure of each component of hydrocarbons. Milkov et al*.*^7^ reported that gas hydrates retrieved at the southern Hydrate Ridge (offshore Oregon) are rich in ethane (C_2_) but exclude propane (C_3_), because the crystal structure I (sI) cannot encage C_3_. Sassen et al*.*^8^ studied the gas hydrates retrieved at the Gulf of Mexico and found that they are high in C_2_, C_3_ and butane (C_4_) but exclude iso-pentane (*i*-C_5_, 2-methylbutane), because the crystal structure II (sII) cannot encage such large guest molecules. Therefore, molecular composition of natural gas primarily controls the crystallographic structure of gas hydrates.

**Figure 1:**
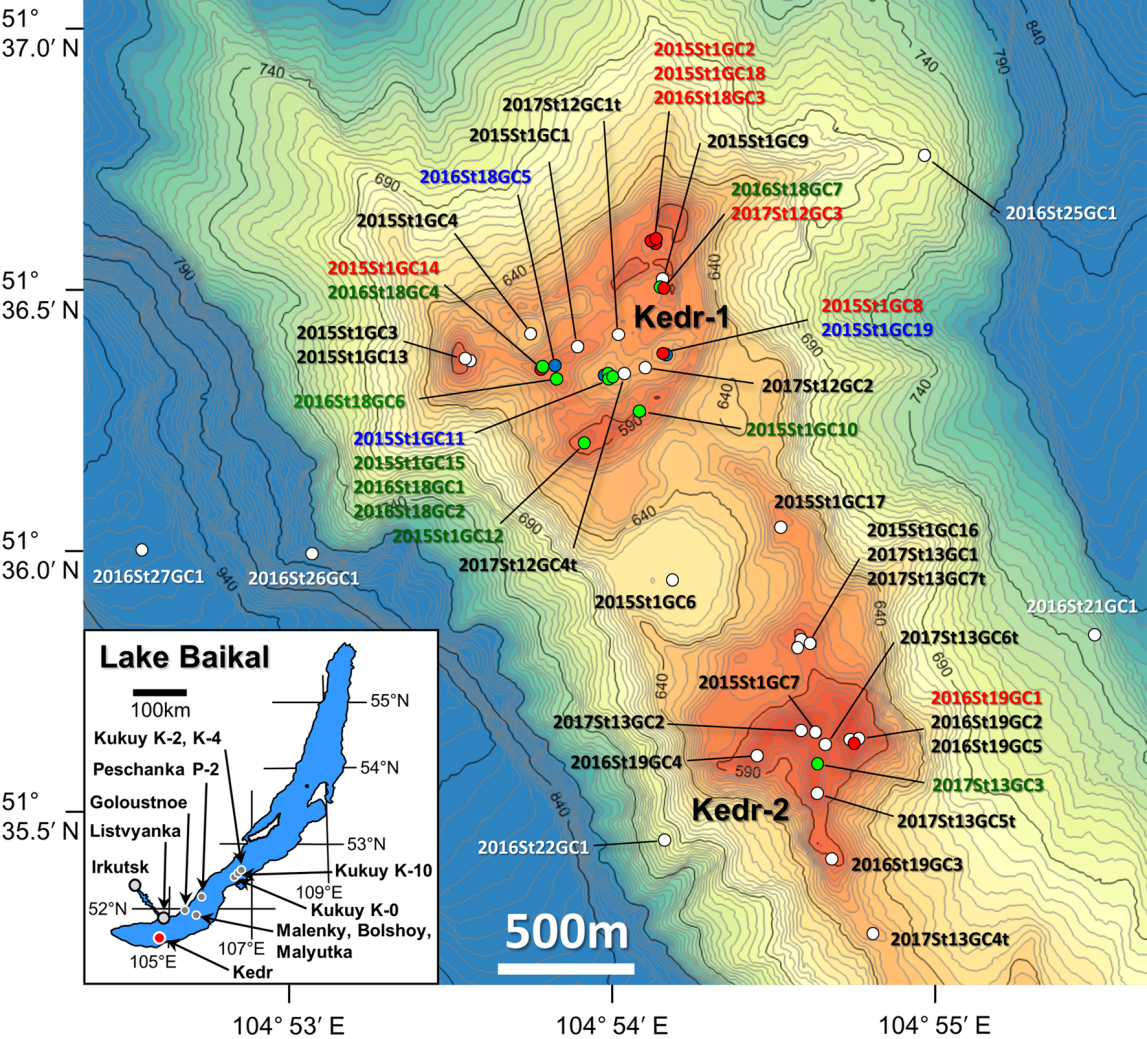
Bathymetry map of the Kedr mud volcano (MV) at the southern basin of Lake Baikal. The blue, red, green and white points indicate structure I only, structure II only, both structures I and II and no gas hydrate, respectively. This map was created using Kashmir 3D, Version 9.3.1 (https://www.kashmir3d.com/).

Natural gas hydrates have been discovered in sub-lacustrine sediments in Lake Baikal, in association with fluid venting at mud volcanoes, pockmarks and cold seeps^9,10^. Although the Lake Baikal gas hydrates generally belong to sI, higher concentrations of C_2_ locally induce the formation of sII as sII gas hydrates form for a particular composition of C_1_ and C_2_^11,12^. Co-existence of sI and sII hydrates in the same sediment core, retrieved at the Kukuy K-2 mud volcano (MV) in the central Baikal basin (Fig. 1), was reported by Kida et al*.*^13,14^. Some formation models for these ‘double structure’ gas hydrates were proposed by Hachikubo et al*.*^15^, Poort et al*.*^16^ and Manakov et al*.*^17^. The hypothesis shared by the latter two papers is that the sII hydrates are formed by re-crystallisation after dissociation of pre-existing sI hydrates. Because C_2_ is prone to be encaged in the hydrate phase rather than C_1_, C_2_ can be concentrated into the hydrate during the re-crystallisation process with C_2_-rich sII forming^18^.

C_2_-rich sII gas hydrates (C_2_ concentration ca. 14% of volatile hydrocarbons) have been discovered not only at the Kukuy K-2 MV but also at the Kukuy K-4 MV and the Kukuy K-10 gas hydrate mound, located in the central Baikal basin, and at the PosolBank seep, located in the southern Baikal basin (Fig. 1)^9,19^. Recently, double-structure gas hydrates were also recovered at the Kedr MV, located in the southern Baikal basin, between 2015 and 2017^10,20^. This study focused on the characteristics of molecular and stable isotope compositions of hydrate-bound hydrocarbons retrieved at the Kedr MV area to improve our understanding of the formation process of double-structure gas hydrates.

The Kedr MV is located in the southern Baikal basin, at 27 km south of Listvyanka (Fig. 1), and it was separated into two study areas: a complex of mud-volcanic buildings (Kedr-1 area), where mud-volcanic breccia was found^20^, and a separate hill with gas hydrate in sediment (Kedr-2 area). Multi-beam echosounder data obtained in 2015 revealed that the Kedr MV consists of small mounds and pockmarks, and results of pore water geochemistry suggested the existence of fluid discharge from greater depths^20^. This part of Lake Baikal is known for the presence of the coal-bearing sediments of the Tankhoy Formation, Oligocene–Miocene age^21,22^. These deposits would represent an ideal source for upward migrating gas that could lead to the formation of gas hydrates near the lake floor^20,22^. During expeditions onboard R/V *G. Yu. Vereshchagin* (VER) in 2015–2017, 42 sediment cores, including 20 hydrate-bearing cores, were sampled at the Kedr MV: samples were taken in August–September 2015 (VER15-03), August 2016 (VER16-03) and August 2017 (VER17-03). Sediment cores were retrieved using gravity corer (length 3.5 or 6.1 m). Coring targets were mainly small mounds and pockmarks, along with five peripheral locations (2016St21, 2016St22, 2016St25, 2016St26 and 2016St27) as references.

## Results

### Gas hydrate crystals

**Figure 2:**
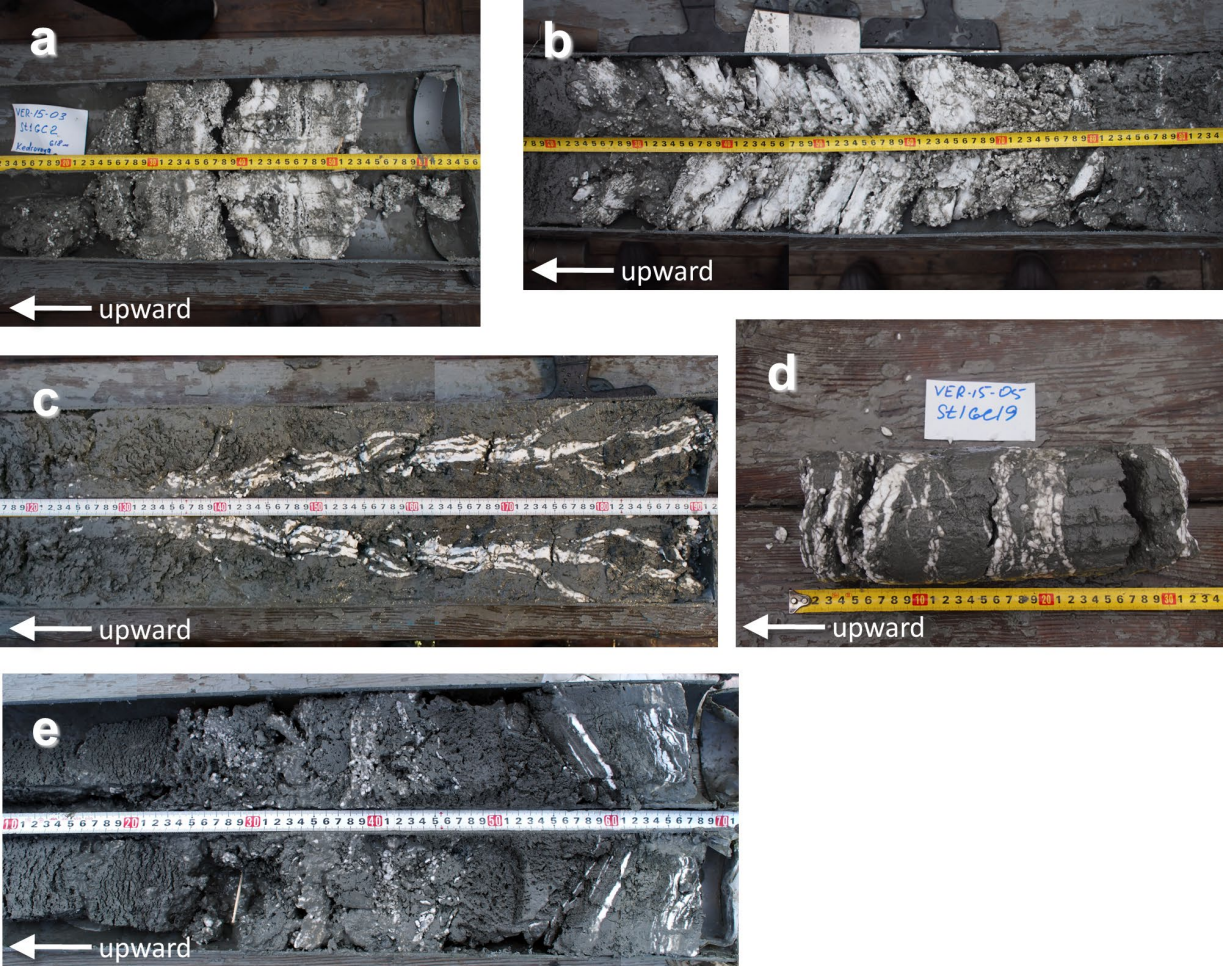
Gas hydrates in sediment cores recovered from the Kedr-1 and Kedr-2 areas. Upward core direction is toward the left in each. (**a)** 2015St1GC2 (Kedr-1, structure II), (**b)** 2015St1GC8 (Kedr-1, structure II), (**c)** 2015St1GC11 (Kedr-1, structure I), (**d)** 2015St1GC19 (Kedr-1, structure I) and € 2017St13GC3 (Kedr-2, upper layer: structure II, lower layer: structure I).

**Figure 3:**
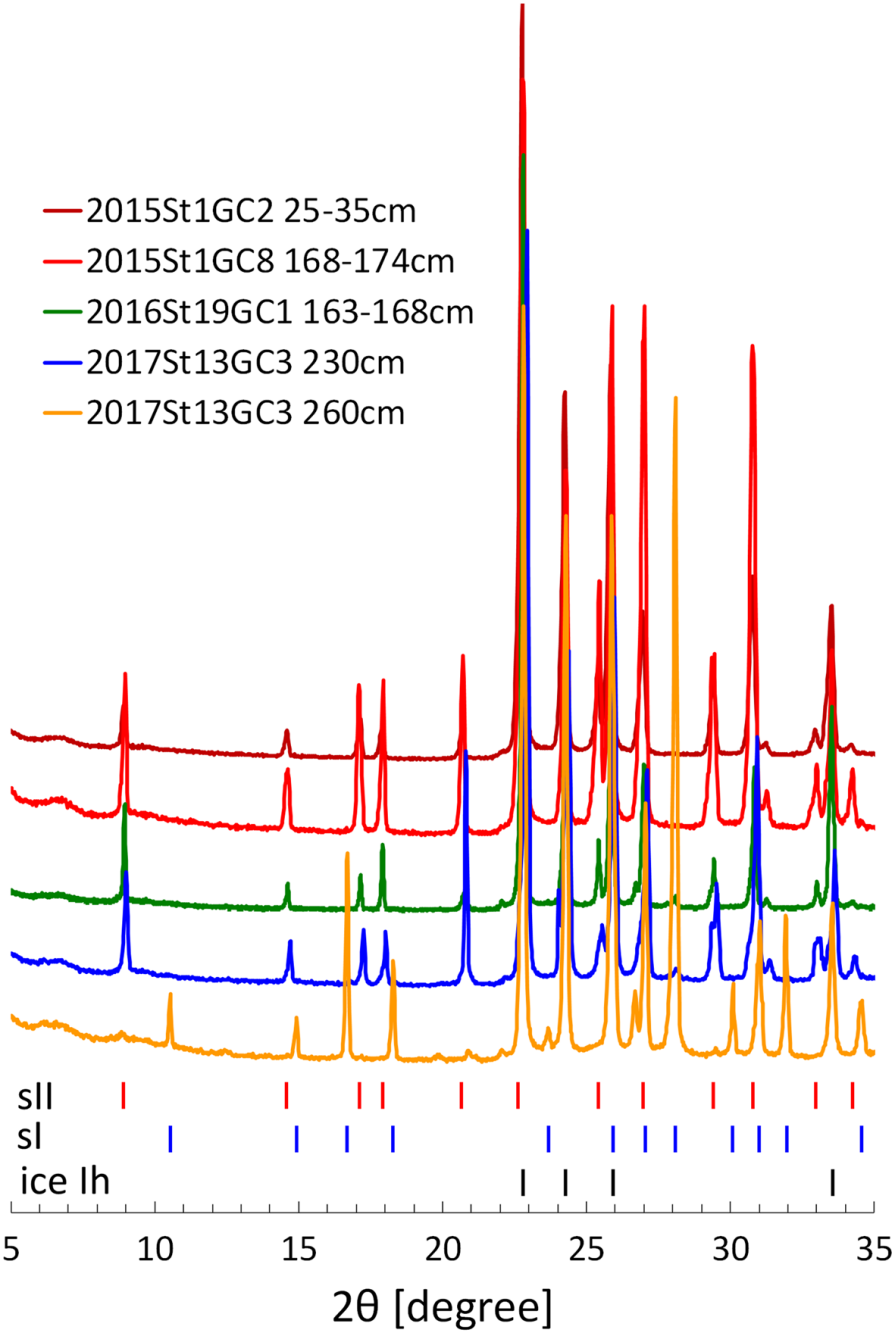
Powder X-ray diffraction profiles of the gas hydrate samples. 2015St1GC2 (25–35 cmblf), 2015St1GC8 (168–174 cmblf), 2016St19GC1 (163–168 cmblf) and 2017St13GC3 (230 cmblf) belong to structure II, whereas 2017St13GC3 (260 cmblf) belongs to structure I. cmblf, centimetres below lake floor.

Gas hydrate crystals in the sediment cores displayed massive, granular, plate-like or vein-like inclusions in the sediments (Fig. 2). Several hydrate-bearing sediment cores had solid granules, suggesting the presence of sII crystals^17^. PXRD profiles of the gas hydrate samples agreed with the crystal shapes (Fig. 3): the granular shape of 2015St1GC2 and the upper layer of 2017St13GC3 (230 cm below lake floor, cmblf) corresponded to sII, whereas plate-like crystals of the lower layer of 2017St13GC3 (260 cmblf) corresponded to sI. Massive crystals of 2015St1GC8, accompanied with granules, corresponded to sII. All samples included ice Ih in the PXRD profiles, caused by partial dissociation of gas hydrates during the retrieval of the cores and handling onboard.

### Molecular and isotopic compositions of hydrate-bound hydrocarbons

**Figure 4:**
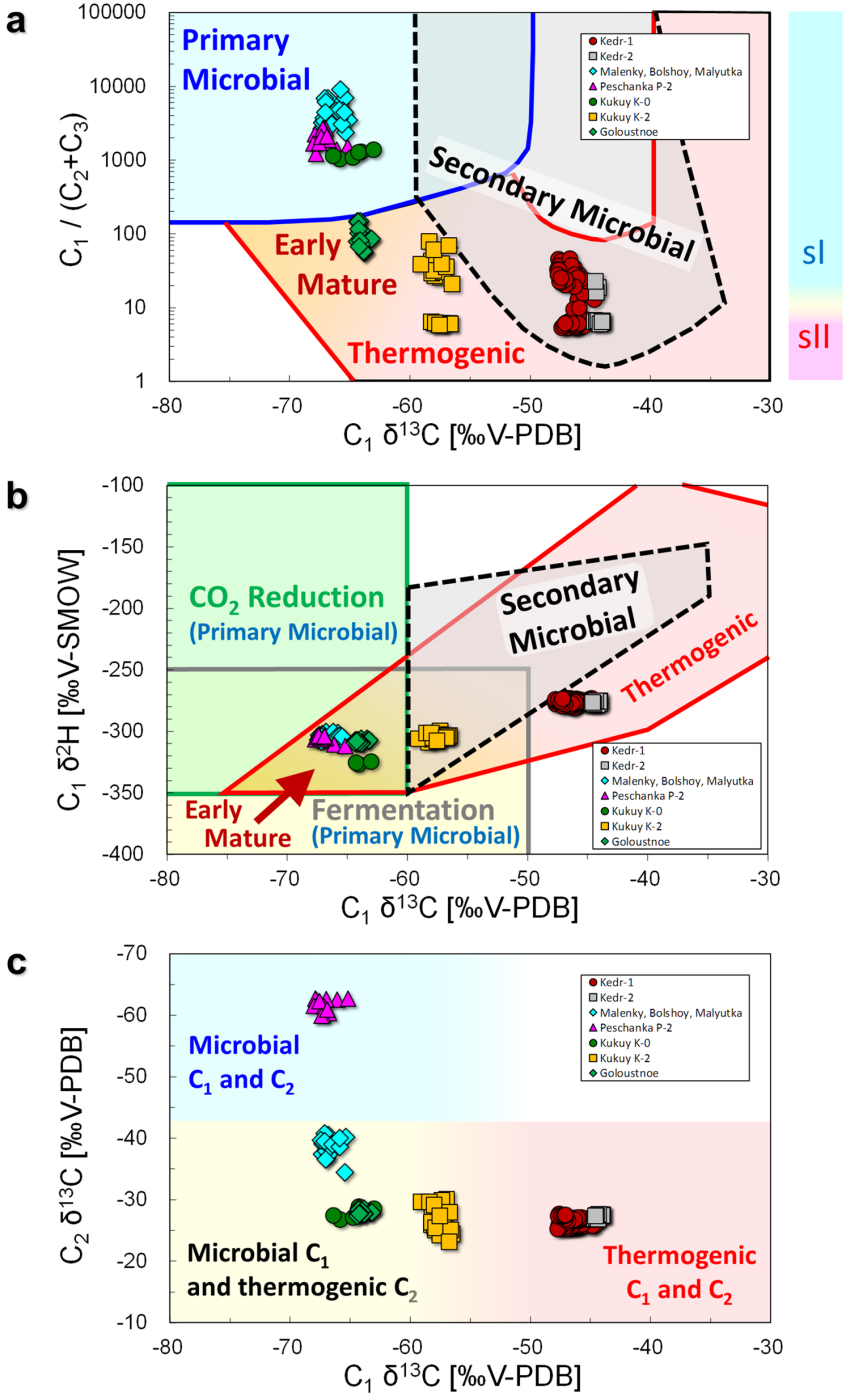
Diagrams of hydrate-bound gases. (**a)** C_1_/(C_2_ + C_3_) plotted against C_1_ δ^13^C, based on the classification of Milkov and Etiope^25^; (**b)** δ^13^C of C_1_ plotted against δ^2^H, based on the classification of Milkov and Etiope^25^; and (**c)** δ^13^C of C_2_ plotted against δ^13^C of C_1_, based on the classification of Milkov^28^. Malenky, Bolshoy, Malyutka, Peschanka P-2, Kukuy K-0, Kukuy K-2 and Goloustnoe data are from Hachikubo et al*.*^23^.

Figure 4 shows the relationship between molecular and isotopic compositions of hydrate- bound hydrocarbons for the different sample sites in Lake Baikal. We obtained 84 samples of hydrate-bound gases from the Kedr-1 area and 12 from the Kedr-2 area. Molecular and isotopic compositions of the hydrocarbons in the hydrate-bound gases are listed in the Supplementary Information and are summarised in Table 1. C_1_ was the main component of the hydrate-bound hydrocarbons, whereas C_2_ was the second component. C_2_ proportions in the hydrocarbons were 2.1–15.6 vol% at Kedr-1 and 4.2–14.1 vol% at Kedr-2. C_3_ proportions were smaller than C_2_ and distributed over a wider range at Kedr-1 (0.0003–0.3039 vol%) and Kedr-2 (0.0035–0.0349 vol%). Therefore, C_1_/(C_2_ + C_3_) in Fig. 4a is mainly influenced by C_1_ and C_2_. C_1_/(C_2_ + C_3_) and C_1_ δ^13^C of the Kedr MV ranged from 5 to 47 and from − 47.8‰ to − 44.0‰, respectively. C_1_/(C_2_ + C_3_) of the Kukuy K-2 MV in the central Baikal basin are clearly separated into two distinct groups responding to the gas hydrate structures: sI (21–80) and sII (~ 6)^23^. Conversely, there was no clear separation in C_1_/(C_2_ + C_3_) values for Kedr-1, suggesting that some gas hydrate samples contained both sI and sII. The relationship between δ^13^C and δ^2^H of C_1_ is shown in Fig. 4b. C_1_ δ^2^H of the Kedr MV was between − 280.5‰ and − 272.8‰, and these values were ~ 30‰ higher than those from other gas hydrate sites. Figure 4c shows an L-shaped distribution between C_1_ δ^13^C and C_2_ δ^13^C in the hydrate-bound gas at Lake Baikal. C_2_ δ^13^C of the Kedr MV ranged from − 27.8‰ to − 25.3‰ and the values were almost the same as Kukuy K-0, Kukuy K-2 and Goloustnoe. Iso-butane (*i*-C_4_, 2-methylpropane), n-butane (*n*-C_4_), neopentane (*neo*-C_5_, 2,2-dimethylpropane) and *i*-C_5_ were detected in the hydrate-bound hydrocarbons (Table 1); however, the concentration of n-pentane (*n*-C_5_) was under the detection limit of our gas chromatograph. The compositions of C_3_, *i*-C_4_ and *neo*-C_5_ detected in the hydrate-bound gases of the sI were smaller than those of sII.

**Table 1:** Molecular and isotopic compositions of hydrate-bound hydrocarbons at the Kedr mud volcano (MV).

Structure	Kedr-1			Kedr-2		
	sI	sI + sII	sII	sI	sI + sII	sII
Number	39	9	36	2	1	9
**Molecular composition [vol%]**						
CH_4_	96.5 ± 0.7	91.8 ± 2.6	85.7 ± 0.7	95.4 ± 0.6	94.2	86.7 ± 0.4
C_2_H_6_	3.5 ± 0.7	8.1 ± 2.6	14.2 ± 0.7	4.6 ± 0.6	5.7	13.3 ± 0.4
C_3_H_8_	0.0041	0.0397	0.0701	0.0073	0.0297	0.0158
*i*-C_4_H_10_	0.0003	0.0034	0.0057	0.0005	0.0039	0.0012
*n*-C_4_H_10_	0.0001	0.0006	0.0007	0.0002	0.0002	0.0002
*neo*-C_5_H_12_	0.0012	0.0095	0.0175	0.0013	0.0119	0.0079
*i*-C_5_H_12_	0.0000	0.0001	0.0001	0.0001	0.0000	0.0000
C_1_/(C_2_ + C_3_)	28.5 ± 6.8	12.3 ± 4.0	6.0 ± 0.4	20.8 ± 2.7	16.4	6.5 ± 0.2
**Isotopic composition [δ**^**13**^**C: V-PDB, δ**^**2**^**H: V-SMOW]**						
C_1_ δ^13^C	− 46.6 ± 0.8	− 45.8 ± 0.5	− 46.5 ± 0.5	− 44.5 ± 0.2	− 44.5	− 44.4 ± 0.3
C_2_ δ^13^C	− 26.5 ± 0.7	− 26.4 ± 0.6	− 26.4 ± 0.6	− 27.5 ± 0.2	− 27.5	− 27.2 ± 0.4
C_3_ δ^13^C	− 10.8 ± 0.9	− 9.9 ± 0.9	− 10.5 ± 0.8	n.d	− 11.6	n.d
C_1_ δ^2^H	− 275.0 ± 1.3	− 274.9 ± 1.2	− 276.9 ± 1.6	− 275.7 ± 1.1	− 277.4	− 276.6 ± 0.9
C_2_ δ^2^H	− 212.5 ± 5.7	− 218.9 ± 7.0	− 216.3 ± 6.2	− 214.9 ± 8.6	− 220.2	− 211.0 ± 2.5

These data list mean values and standard deviations, sorted into three groups: sI, where C_2_ composition was < 5%; sI + sII, where C_2_ composition was 5%–13%; and sII, where C_2_ composition was > 13%. n.d., not determined.

### Molecular and isotopic compositions of sediment gases

**Figure 5:**
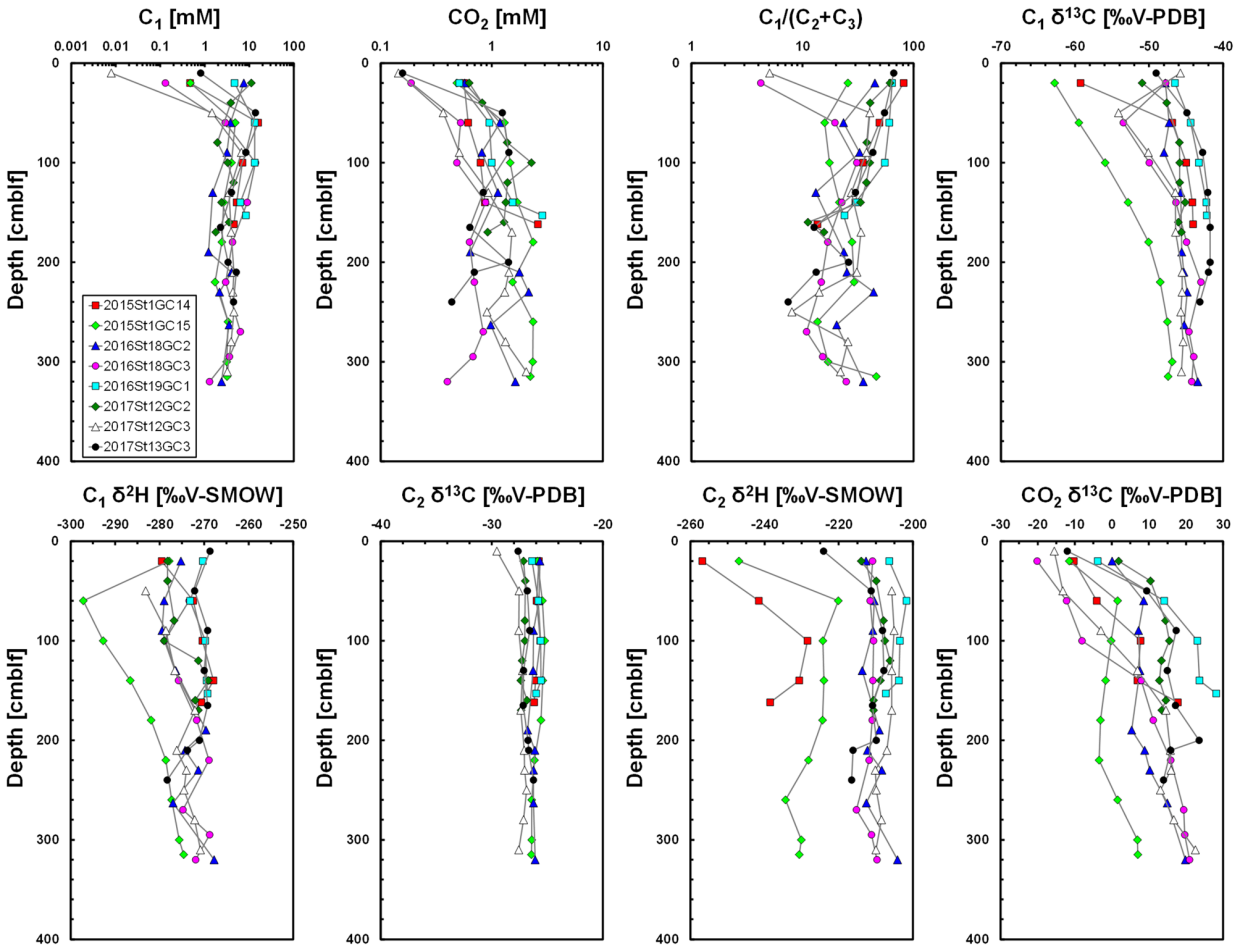
Depth profiles of C_1_ and CO_2_ concentrations, C_1_/(C_2_ + C_3_) values, C_1_ δ^13^C, C_1_ δ^2^H, C_2_ δ^13^C, C_2_ δ^2^H and CO_2_ δ^13^C in the headspace gas. cmblf, centimetres below lake floor.

Figure 5 shows the selected depth profiles of sediment gas in the hydrate-bearing cores obtained using headspace gas method (all data are shown in the Supplementary Information). C_1_ concentrations of the all sediment cores increased with depth and reached 1–10 mM at a depth of around 50 cm below lake floor (cmblf). The CO_2_ concentrations of all cores increased slightly with depth. The value of C_1_/(C_2_ + C_3_) generally decreased with depth. Because C_3_ concentrations in the sediment gases were three orders of magnitude smaller than C_2_ concentrations (average concentrations of C_2_ and C_3_ for all sediment gases are 146 μM and 0.13 μM, respectively), these results indicated that the Kedr MV area with gas hydrates is characterised by a high C_2_ concentration.

C_1_ δ^13^C of all cores increased with depth; however, it also increased beneath the lake floor (10–20 cmblf) in 2016St18GC3 and 2017St12GC3 cores, where C_1_ concentrations were low, suggesting oxidation of C_1_ and consumption of light C_1_. C_1_ δ^13^C of Kedr-1 and Kedr-2 was around − 45‰ and − 42‰, respectively. C_1_ δ^2^H of hydrate-bearing cores was between − 280‰ and − 270‰ although that of 2015St1GC15 was between − 300‰ and − 270‰. C_2_ δ^13^C of all cores was almost constant with depth and averaged at around − 26‰, suggesting thermogenic C_2_. C_2_ δ^2^H of hydrate-bearing cores was at around − 210‰; however, that of some sediment cores (2015St1GC14 and 2015St1GC15) was between − 240‰ and − 230‰ at their base. CO_2_ δ^13^C of all cores generally increased with depth, reaching + 20‰ (Kedr-1) and + 30‰ (Kedr-2).

## Discussion

### Origin of hydrate-bound hydrocarbons

A relationship between C_1_/(C_2_ + C_3_) and C_1_ δ^13^C has been applied to identify the sources of hydrocarbons in submarine seeps^24^. Recently, this diagram was revised based on a large dataset^25^. As shown in Fig. 4a, hydrate-bound hydrocarbons at the Kedr MV have thermogenic and/or secondary microbial origins, whereas those of other gas hydrate sites (Malenky, Bolshoy, Malyutka, Peschanka P-2, Kukuy K-0, Kukuy K-2 and Goloustnoe; Fig. 1) in Lake Baikal demonstrate microbial or early mature thermogenic origins. The hydrate-bound C_1_ from all locations except those at the Kedr MV were interpreted to be of microbial origin via methyl-type fermentation^23^ according to Whiticar’s old diagram^26^; however, the revised diagram^25^ suggests early mature thermogenic gases (Fig. 4b). Those of the Kedr MV plot at the boundary of the thermogenic and secondary microbial origin zones. Low C_1_ and C_2_ δ^13^C at the Peschanka P-2 MV indicated that C_1_ and C_2_ are of microbial origin^27,28^, whereas Kedr MV shows high C_1_ and C_2_ δ^13^C indicating their thermogenic origin (Fig. 4c). At other sites, C_1_ and C_2_ δ^13^C suggested that gases are mainly of microbial origin (in terms of C_1_) with some thermogenic component (^13^C rich and higher concentration in C_2_).

Stable isotopes in hydrate-bound C_1_ at the Kedr-1 and Kedr-2 areas suggested its thermogenic origin. However, it is close to the field of secondary microbial C_1_ in Fig. 4b, and the data are plotted in the overlap between the fields of thermogenic and secondary microbial in Fig. 4a. Milkov^29^ mentioned that secondary microbial C_1_ is characterised by C_1_-rich dry gas, large C_1_ δ^13^C (between − 55‰ and − 35‰) and large CO_2_ δ^13^C (more than + 2 ‰). Although hydrate-bound and sediment gases in the Kedr MV were not C_1_ rich and contained 3%–15% of C_2_, C_1_ δ^13^C was around − 45‰, which agrees with the secondary microbial C_1_. Because some data of secondary microbial gas are plotted outside the field on the original graph^25^, we could include the gas data in the category of secondary microbial C_1_ in Fig. 4b.

**Figure 6:**
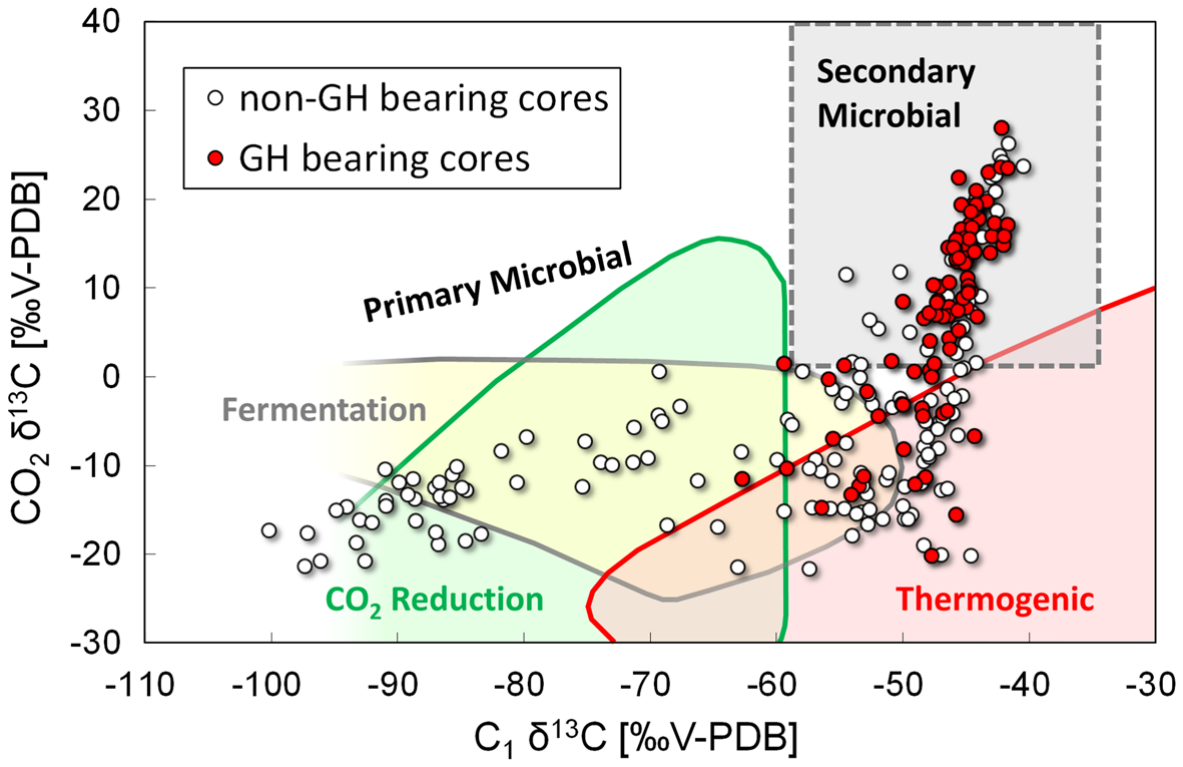
A diagram of headspace gases. CO_2_ δ^13^C plotted against C_1_ δ^13^C, based on the classification of Milkov and Etiope^25^.

Figure 6 shows the relationship between C_1_ δ^13^C and CO_2_ δ^13^C in the sediment gas obtained using headspace gas method. According to the genetic diagram^25^, gas hydrate cores are plotted at the zones of the thermogenic and secondary microbial origins, whereas the cores at the peripheral area are primary microbial. The headspace gas data of the hydrate-bearing cores in Fig. 6 seem to be plotted in the field of thermogenic gas (low CO_2_ δ^13^C), but the effect of light CO_2_ produced by methane oxidation in the subsurface layer also decreased CO_2_ δ^13^C as shown in Fig. 5. These results suggested that secondary microbial C_1_ mixes into thermogenic gas. Coal-bearing sediments exist around the Kedr area^21,22^, and secondary microbial C_1_ can also form from coal beds^30^. Hydrate-bound C_1_ of secondary microbial origin has been only reported at the Alaska North Slope^31^. This study is another case for it.

### Formation process of the sII gas hydrates

As stated before, the crystallographic structure of gas hydrates at the Kedr MV is mainly due to the composition of thermogenic C_2_ in the volatile hydrocarbons. The concentration of C_3_, which is one of the sII-forming components, was two to three orders of magnitude smaller than that of C_2_, because biodegradation occurs and this preferentially reduces C_3−5_ of *n*-alkanes^19,32, 33^. The concentration of *n*-C_4_ was smaller than that of *i*-C_4_, whereas that of *n*-C_5_ was not detected (Table 1). C_3_ δ^13^C was around − 10‰, suggesting that light C_3_ is consumed by microbial activity. Assuming that sediment gas C_3+_ can be ignored, sediment gas ratio C_1_/C_2_ at the study area was 30 ± 17 (mean and standard deviation), and the concentration of C_2_ was ~ 3%. Such a composition of thermogenic gas is, therefore, considered to be supplied from a deep sediment layer, forming sI gas hydrates composed of mainly C_1_ and C_2_^11,12^ in the lake floor sediment.

In the cases where sI gas hydrates plug and block migration pathways, upward fluid flow becomes more focused in other areas^16^. Once gas supply stops locally, gas hydrates begin to decompose, with the gas dissolving into gas–poor sediment pore water. In the system of C_1_ and C_2_, C_2_ is prone to be encaged in gas hydrate and decreases the equilibrium pressure of mixed-gas hydrate. Therefore, C_2_-rich gas hydrate forms in parallel with the decomposition of sI gas hydrate. The Colorado School of Mines Hydrate (CSMHYD) program^34^ showed that C_2_-rich sII gas hydrate (C_2_ concentration 17%) forms from mixed gas composed of C_1_ and C_2_ (C_2_ concentration 3%). The C_2_ concentration of hydrate-bound gas at the Kedr MV was ~ 14%, agreeing fairly well with the results of the CSMHYD program. Such secondary generation of gas hydrates can produce compositions and crystallographic structures that are different from the original crystals. A calorimetric study of synthetic C_1_ and C_2_ mixed-gas hydrate revealed that double peaks of heat flow correspond to the dissociation process of C_1_ and C_2_ mixed-gas hydrate, suggesting that C_2_-rich gas hydrate forms simultaneously from dissociated gas and showed that the second heat flow peak correspond to the dissociation of C_2_-rich gas hydrate^18^. The PXRD and solid-state ^13^C nuclear magnetic resonance techniques demonstrated that C_2_-rich sI gas hydrate forms in the dissociation process of C_1_ + C_2_ sII gas hydrate^35^.

Among twenty hydrate-bound cores in the Kedr area, four cores contained sI only, seven cores had sII only, and seven cores showed sII at the upper layer and sI at the lower layer, as observed at the Kukuy K-2 MV^13,16,17^. Furthermore, in the cores 2015St1GC15 and 2016St18GC2, gas hydrate structure had sI at the upper and lower layer, and sII at the middle layer. These results suggested that complex gas hydrate layers are composed of sI and sII in subsurface sediments as shown in the schematic illustration in Poort et al*.*^16^.

**Figure 7:**
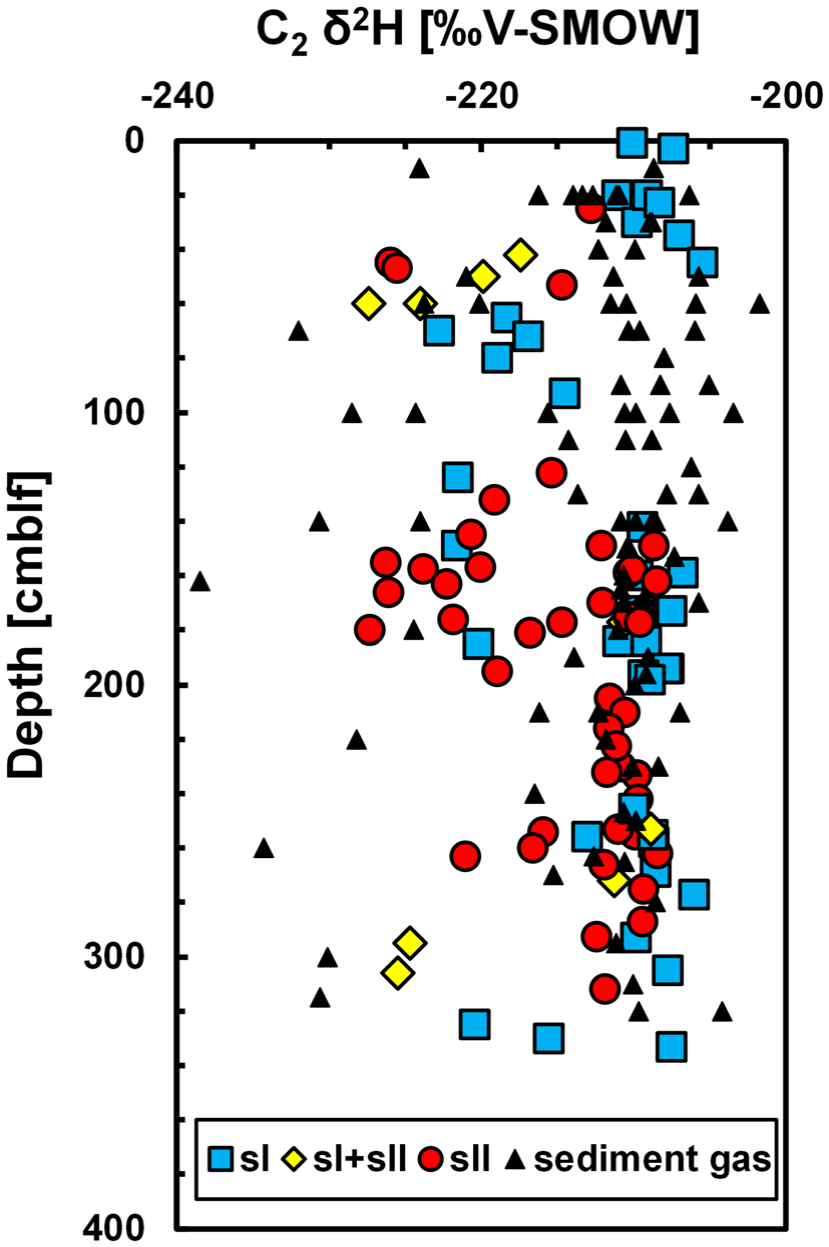
Depth profiles of C_2_ δ^2^H of hydrate-bound and sediment gases. cmblf, centimetres below lake floor.

Depth profiles of C_2_ δ^2^H of gas hydrate cores from the Kedr MV are shown in Fig. 7. C_2_ δ^2^H of hydrate-bound gases varied between − 227‰ and − 206‰, with a grouping around − 210‰. C_2_ δ^2^H of sediment gases was also around − 210‰, indicating that C_2_ δ^2^H of the original thermogenic gas is − 210‰. As stated above, C_2_ δ^2^H of some cores showed low values at their base. Based on the isotopic fractionation of hydrogen in C_2_ during the formation of sI C_2_ hydrate^36^, δ^2^H of hydrate-bound C_2_ was 1.1‰ lower than that of residual C_2_. However, this is too small to explain the wide distribution in C_2_ δ^2^H shown in Fig. 7. On the other hand, Matsuda et al*.*^37^ reported that isotopic fractionation of hydrogen in C_2_ is dependent on the crystallographic structure: 1‰–2‰ for sI and ~ 10‰ for sII. Gas hydrates plotting around − 220‰ in C_2_ δ^2^H can be explained as a secondary generation of sII from dissociated gas hydrates, of which C_2_ δ^2^H was around − 210‰. However, some sII samples showed high C_2_ δ^2^H (around − 210‰), whereas some sI samples showed low C_2_ δ^2^H (around − 220‰). These results indicated that formation and dissociation processes of gas hydrates produce complicated isotopic profiles in C_2_ δ^2^H under non-equilibrium conditions.

### Characteristics of hydrate-bound gases in sII

**Figure 8:**
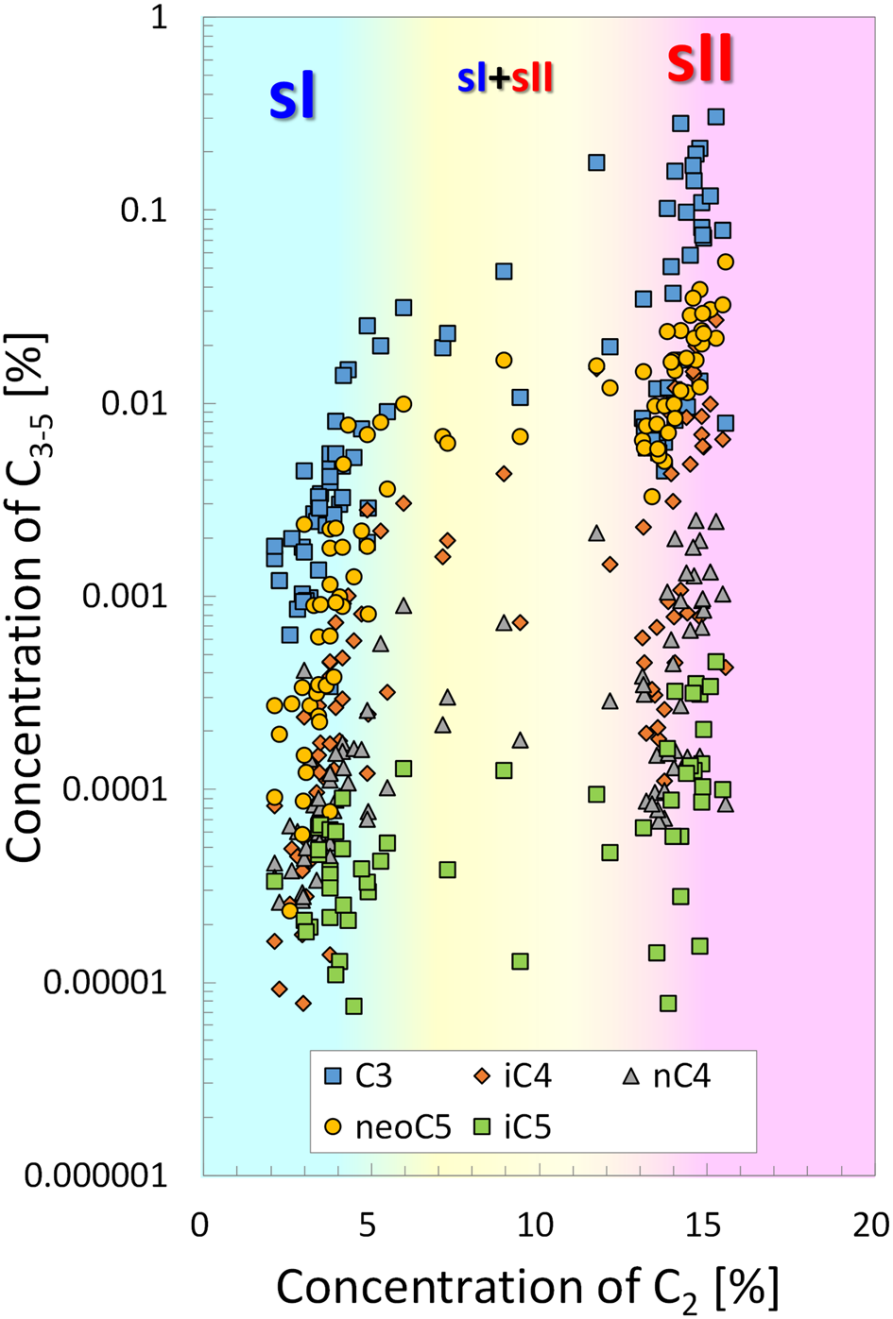
Concentration of C_3–5_ against C_2_ concentration in the hydrate-bound gases.

C_3_, *i*-C_4_, *n*-C_4_ and *neo*-C_5_ can be encaged in the larger hexadecahedral cages of sII^1^. *n*-C_4_ and *neo*-C_5_ can be encaged using a help gas (e.g. C_1_) to fill in the smaller dodecahedral cages of sII, because they cannot form pure *n*-C_4_ and *neo*-C_5_ hydrates, respectively. Figure 8 shows the concentration of C_3_, *i*-C_4_, *n*-C_4_, *neo*-C_5_ and *i*-C_5_ plotted against C_2_ concentration. The figure illustrates a clear division between sI (3–4%) and sII (14%) C_2_ concentrations. Data points between C_2_ concentrations of 5% and 13% were considered to have a mixture of sI and sII. Concentrations of C_3_, *i*-C_4_, *n*-C_4_ and *neo*-C_5_ had a positive correlation with the concentration of C_2_, and these concentrations in sII were 1 or 2 orders of magnitude larger than those in sI, suggesting that C_3_, *i*-C_4_, *n*-C_4_ and *neo*-C_5_ are encaged with C_2_ in the sII formation process.

C_3_ values of 0.001%–0.01%, ~ 0.0001% of *n*-C_4_, and 0.0001%–0.01% of *neo*-C_5_ were also detected in sI hydrate-bound gas (Fig. 8), despite these hydrocarbons being unable to be encaged in sI. This can be explained by gases being adsorbed with sediments and gas hydrate crystals, which are then trapped in the grain boundary of polycrystalline gas hydrate crystals, and the gases are encaged if a small amount of sII crystals are present. For example, Uchida et al*.*^38^ examined natural gas hydrate retrieved at the Mackenzie Delta (onshore Canada) and detected C_3_ encaged in sII using Raman spectroscopy, although PXRD results suggested that the sample was sI and the major component of hydrate-bound gas was C_1_ (more than 99%).

*neo*-C_5_ is considered to form from the decomposition of gem-dimethylcycloalkanes derived from the terpenes of terrestrial organic matter^39^. It is easily enriched by preferential diffusion due to the nearly spherical molecules and its diffusion coefficient, which is higher than that of less branched isomers^40^. The sII hydrates retrieved at the Kukuy K-2 MV (central Baikal basin) contained 0.026–0.064% of *neo*-C_5_ in the volatile hydrocarbons^13,14^, and those at the Kedr MV had a maximum value of 0.054% of *neo*-C_5_ (Supplementary Information Table S1). On the contrary, in the case of natural gas hydrates retrieved at the Joetsu Basin (Japan Sea), *neo*-C_5_ was excluded and remained in sediment during the formation of sI gas hydrates from C_1_-rich gas^41^. The molecular size of *i*-C_5_ is considerably large to be encaged in the large cages of sII. Maximum concentration of *i*-C_5_ in the hydrate-bound gases was in several parts per million in both the fields of sI and sII (Fig. 8), indicating that *i*-C_5_ is not a hydrate-bound hydrocarbon and adsorbed with gas hydrate crystals and/or trapped in their grain boundary.

## Conclusion

We reported the molecular and stable isotope compositions of hydrate-bound and sediment gases at the Kedr MV in the southern Baikal basin. The empirical classifications of the molecular and isotopic compositions of hydrate-bound hydrocarbons showed that the gas source is mainly thermogenic, one of the end members of hydrate-bound gases in Lake Baikal. Large CO_2_ δ^13^C in the sediment gases suggested that secondary microbial C_1_ mixes with thermogenic gas. Double-structure gas hydrates composed of sI and sII were observed, likely created by sI crystals partly dissociating and C_2_-rich sII crystals forming. The C_2_ δ^2^H values of hydrate-bound gas revealed that light C_2_ is preferentially encaged into sII crystals; however, some exceptions indicated that more complicated processes of gas exchange might exist between sI, sII and the dissolved gas in pore water. Because C_2_ is preferentially concentrated into the gas hydrate phase, high concentration of thermogenic C_2_ produce sII crystals with C_1_, and C_2_ is encaged into the large cages of sII with C_3_, *i*-C_4_, *n*-C_4_, and *neo*-C_5_ in the re-crystallisation process.

## Methods

Gas hydrate crystals were collected onboard R/V *G. Yu. Vereshchagin* and stored in liquid nitrogen. Powder X-ray diffraction (PXRD) measurements were performed to check the crystallographic structure. Samples for PXRD were finely ground at a temperature of 77 K and then kept at 173 K. The X-ray diffraction patterns were recorded at 2θ = 5 − 35° using Cu Kα radiation (λ = 1.5418 Å) and a Bruker D8 Advance diffractometer equipped with a TTK 450 Anton Paar temperature controlling device. The positions of diffraction peaks corresponding to sI and sII hydrates and hexagonal ice (Ih) were calculated with the use of reference data on space group and unit cell parameters of the respective compounds^42^.

Hydrate-bound gases were collected using water displacement method and stored in 5 mL glass vials sealed with butyl septum stoppers. To avoid microbial alteration, 0.3 mL of preservative (50 wt% aqueous solution of benzalkonium chloride) was introduced into the vials. Gas sampling was conducted for each layer of gas hydrate in the hydrate-bearing cores. Several vials of samples were taken from a hydrate nodule. Sediment gases were collected using headspace gas method to calculate the depth profiles of each gas component in the sediment cores. To create a 5 mL headspace, 10 mL of sediment and 10 mL of saturated aqueous solution of NaCl were introduced into 25 mL-glass vials. The headspace was flushed with helium, the carrier gas used in the gas chromatography, to reduce air contamination^43^. The headspace gases were then placed into the 5 mL glass vials to separate them from sediment particles or water and to prevent any microbial activity during storage.

The molecular compositions of the hydrocarbons (from C_1_ to C_5_) were determined using a gas chromatograph (GC-2014, Shimadzu, Kyoto, Japan) equipped with a packed column (Shimadzu Sunpak-S; length 2 m, inner diameter [ID] 3 mm), along with a thermal conductivity detector and flame ionisation detector for detecting high and low concentrations of hydrocarbons respectively. The two detectors were connected in series. The detection limit was 0.5 ppmv (C_1_–C_3_) and 5 ppmv (C_4_–C_5_). The analytical error estimated by multiple injections of standard gases was < 1.2% for each gas component. Stable carbon and hydrogen isotopic ratios of the hydrocarbons and CO_2_ were measured using a continuous-flow isotope-ratio mass spectrometer (CF-IRMS, DELTA V, Thermo Fisher Scientific, Waltham, MA, USA) coupled with a gas chromatograph (TRACE GC Ultra, Thermo Fisher Scientific). The gas chromatograph was equipped with a Carboxen-1006 PLOT capillary column (length 30 m, ID 0.32 mm, film thickness 15 μm, Sigma-Aldrich, St. Louis, MO, USA). In the case of samples with low C_1_ concentration, a Sigma-Aldrich Carboxen-1010 PLOT capillary column (length 30 m, ID 0.32 mm, film thickness 15 μm) was also used to separate air components from C_1_. Stable isotope compositions were reported as δ values (in per-mille):

1 $$\updelta [\permil ]=\left(\frac{{R}_{sample}-{R}_{standard}}{{R}_{standard}}\right)\times 1000$$

where R denotes the ^13^C/^12^C or ^2^H/^1^H ratio. δ^13^C and δ^2^H are given with reference to the V-PDB and V-SMOW standards, respectively, determined using NIST RM8544 (NBS19) for δ^13^C and NIST RM8561 (NGS3) for δ^2^H. The analytical precision was 0.3‰ for hydrocarbon (C_1_–C_3_) δ^13^C and 1‰ for δ^2^H.

## Supplementary information

Supplementary information.

## Data Availability

All the gas data are reported in the Supplementary Information.
