# Supplementary material for: Characteristics of hydrate-bound gas retrieved at the Kedr mud volcano (southern Lake Baikal)
Source: Sci Rep. 2020 Sep 8;10:14747. doi: 10.1038/s41598-020-71410-2 (PMC7479611; doi:10.1038/s41598-020-71410-2)
Supplement: Supplementary file 1 — Supplementary information. [file 41598_2020_71410_MOESM1_ESM.pdf]

Supplementary Information

Characteristics of hydrate-bound gas retrieved at the Kedr mud volcano (southern Lake Baikal)

Akihiro Hachikubo[1], Hirotsugu Minami[1], Satoshi Yamashita[1], Andrey Khabuev[2], Alexey Krylov[2,3,4], Gennadiy Kalmychkov[5], Jeffrey Poort[6], Marc De Batist[7], Alexandr Chenskiy[8], Andrey Manakov[9], and Oleg Khlystov[2]

[1] Kitami Institute of Technology, 165 Koen-cho, Kitami 090-8507, Japan
[2] Limnological Institute, SB RAS, 3 Ulan-Batorskaya St., Irkutsk 664033, Russia
[3] Institute of Earth Sciences, St. Petersburg State University, 7-9, Universitetskaya nab., 199034 St. Petersburg, Russia
[4] VNIIOkeangeologia, Anglyisky prospect 1, 190121 St. Petersburg, Russia
[5] Vinogradov Institute of Geochemistry, SB RAS, 1-a Favorsky St., Irkutsk 664033, Russia
[6] Sorbonne Université, CNRS, Institut des Sciences de la Terre de Paris, ISTeP, 4 place Jussieu, F-75005 Paris, France
[7] Renard Centre of Marine Geology, Ghent University, Krijgslaan 281 s8, 9000 Ghent, Belgium
[8] Irkutsk National Research Technical University, 83 Lemontov St., Irkutsk 664074, Russia
[9] Nikolaev Institute of Inorganic Chemistry, SB RAS, 3 Acad. Lavrentiev Ave., Novosibirsk 630090, Russia

Corresponding author: Akihiro Hachikubo (hachi@mail.kitami-it.ac.jp)

Table S1. Molecular and isotopic compositions of hydrate-bound hydrocarbons at the Kedr mud volcano located in the southern Baikal basin. Crystallographic structure of gas hydrate is estimated as structure I (sI), where ethane (C_2) composition was <5%; structure II (sII), where C_2 composition was >13%; and sI+sII, where C_2 composition was 5%–13%. cmblf, centimetres below lake floor. n.d., not determined

Table S2. Molecular and isotopic compositions of headspace gases at the Kedr mud volcano. cmblf, centimetres below lake floor. n.d., not determined

Table S1

								Molecular Composition									Isotopic Composition					Crystallographic Structure
Date	Cruise Name	Core Name	Latitude [°N]	Longitude [°E]	Water Depth [m]	Sample Depth [cmblf]	Place	CH_4 [%]	C_2H_6 [%]	C_3H_8 [%]	i-C_4H_{10} [%]	n-C_4H_{10} [%]	neo-C_5H_{12} [%]	i-C_5H_{12} [%]	n-C_5H_{12} [%]	$C_1/(C_2+C_3)$	CH_4 $\delta^{13}C$ [‰V-PDB]	C_2H_6 $\delta^{13}C$ [‰V-PDB]	C_3H_8 $\delta^{13}C$ [‰V-PDB]	CH_4 δ^2H [‰V-SMOW]	C_2H_6 δ^2H [‰V-SMOW]	
2015/8/16	VER15-03	2015St1GC2	51.3635	104.5408	618	25	Kedr-1	86.9	13.1	0.0084	0.0006	0.0004	0.0064	0.0000	0.0000	7	-46.0	-25.8	n.d.	-276.9	-212.8	II
2015/8/16	VER15-03	2015St1GC2	51.3635	104.5408	618	47	Kedr-1	85.0	14.8	0.2084	0.0221	0.0020	0.0388	0.0003	0.0000	6	-45.5	-25.7	-9.5	-276.6	-225.5	II
2015/8/16	VER15-03	2015St1GC2	51.3635	104.5408	618	53	Kedr-1	86.9	13.1	0.0075	0.0005	0.0003	0.0059	0.0000	0.0000	7	-46.3	-25.7	n.d.	-276.2	-214.7	II
2015/8/16	VER15-03	2015St1GC8	51.3623	104.5410	625	132	Kedr-1	85.0	14.8	0.1098	0.0085	0.0010	0.0204	0.0001	0.0000	6	-47.2	-25.9	-10.5	-276.9	-219.2	II
2015/8/16	VER15-03	2015St1GC8	51.3623	104.5410	625	145	Kedr-1	85.0	14.8	0.0814	0.0069	0.0007	0.0237	0.0001	0.0000	6	-47.1	-25.7	-11.2	-276.7	-220.7	II
2015/8/16	VER15-03	2015St1GC8	51.3623	104.5410	625	158	Kedr-1	85.1	14.7	0.1946	0.0198	0.0025	0.0168	0.0004	0.0000	6	-46.5	-25.9	-11.5	-275.8	-223.8	II
2015/8/16	VER15-03	2015St1GC8	51.3623	104.5410	625	163	Kedr-1	84.4	15.3	0.3039	0.0269	0.0024	0.0218	0.0005	0.0000	5	-46.9	-25.9	-11.2	-274.2	-222.3	II
2015/8/16	VER15-03	2015St1GC8	51.3623	104.5410	625	177	Kedr-1	85.2	14.6	0.1429	0.0140	0.0013	0.0216	0.0001	0.0000	6	-47.1	-26.0	-11.1	-276.4	-214.7	II
2015/8/16	VER15-03	2015St1GC8	51.3623	104.5410	625	195	Kedr-1	84.4	15.6	0.0079	0.0004	0.0001	0.0540	0.0000	0.0000	5	-47.4	-25.3	n.d.	-276.0	-219.0	II
2015/8/16	VER15-03	2015St1GC10	51.3616	104.5405	609	42	Kedr-1	94.5	5.5	0.0090	0.0003	0.0001	0.0036	0.0001	0.0000	17	-46.0	-25.5	n.d.	-274.2	-217.4	I+II
2015/8/16	VER15-03	2015St1GC10	51.3616	104.5405	609	50	Kedr-1	92.9	7.1	0.0194	0.0016	0.0002	0.0068	0.0000	0.0000	13	-44.7	-25.8	-9.6	-272.8	-219.9	I+II
2015/8/16	VER15-03	2015St1GC10	51.3616	104.5405	609	65	Kedr-1	95.1	4.9	0.0029	0.0002	0.0001	0.0008	0.0000	0.0000	19	-44.9	-25.8	n.d.	-273.7	-218.3	I
2015/8/16	VER15-03	2015St1GC11	51.3620	104.5359	636	124	Kedr-1	96.8	3.2	0.0010	0.0000	0.0001	0.0003	0.0000	0.0000	30	-46.6	-25.6	n.d.	-275.1	-221.5	I
2015/8/16	VER15-03	2015St1GC11	51.3620	104.5359	636	149	Kedr-1	97.0	3.0	0.0045	0.0002	0.0004	0.0024	0.0000	0.0000	32	-46.5	-25.4	n.d.	-274.2	-221.6	I
2015/8/16	VER15-03	2015St1GC11	51.3620	104.5359	636	185	Kedr-1	96.7	3.3	0.0027	0.0001	0.0001	0.0009	0.0000	0.0000	29	-46.9	-25.6	n.d.	-274.6	-220.2	I
2015/8/24	VER15-03	2015St1GC12	51.3613	104.5355	604	45	Kedr-1	86.5	13.5	0.0066	0.0003	0.0001	0.0097	0.0000	0.0000	6	-46.5	-26.2	n.d.	-274.9	-226.0	II
2015/8/24	VER15-03	2015St1GC12	51.3613	104.5355	604	45	Kedr-1	86.3	13.7	0.0063	0.0003	0.0001	0.0097	0.0000	0.0000	6	-46.5	-26.1	n.d.	-275.7	-226.0	II
2015/8/24	VER15-03	2015St1GC12	51.3613	104.5355	604	unknown	Kedr-1	96.6	3.4	0.0026	0.0001	0.0001	0.0006	0.0001	0.0000	28	-46.8	-25.9	n.d.	-274.0	-221.0	I
2015/8/24	VER15-03	2015St1GC12	51.3613	104.5355	604	unknown	Kedr-1	96.5	3.5	0.0034	0.0002	0.0001	0.0009	0.0001	0.0000	28	-46.5	-25.7	n.d.	-274.1	-223.1	I
2015/8/24	VER15-03	2015St1GC12	51.3613	104.5355	604	unknown	Kedr-1	95.1	4.9	0.0019	0.0001	0.0001	0.0018	0.0000	0.0000	20	-44.9	-25.8	n.d.	-274.5	-223.9	I
2015/8/24	VER15-03	2015St1GC14	51.3621	104.5347	625	155	Kedr-1	86.3	13.7	0.0045	0.0001	0.0001	0.0050	0.0000	0.0000	6	-46.1	-25.6	n.d.	-276.9	-226.3	II
2015/8/24	VER15-03	2015St1GC14	51.3621	104.5347	625	166	Kedr-1	86.4	13.6	0.0055	0.0002	0.0001	0.0054	0.0000	0.0000	6	-46.9	-25.8	n.d.	-277.1	-226.1	II
2015/8/24	VER15-03	2015St1GC14	51.3621	104.5347	625	176	Kedr-1	86.5	13.5	0.0059	0.0002	0.0001	0.0058	0.0000	0.0000	6	-46.1	-25.8	n.d.	-275.9	-221.9	II
2015/8/24	VER15-03	2015St1GC14	51.3621	104.5347	625	180	Kedr-1	86.8	13.2	0.0059	0.0002	0.0001	0.0076	0.0000	0.0000	7	-46.4	-25.9	n.d.	-276.2	-227.3	II
2015/8/24	VER15-03	2015St1GC15	51.3620	104.5400	638	256	Kedr-1	97.1	2.9	0.0018	0.0000	0.0000	0.0003	0.0000	0.0000	33	-47.0	-25.4	n.d.	-275.1	-213.0	I
2015/8/24	VER15-03	2015St1GC15	51.3620	104.5400	638	295	Kedr-1	92.7	7.3	0.0230	0.0019	0.0003	0.0062	0.0000	0.0000	13	-46.1	-25.9	-10.3	-276.0	-224.7	I+II
2015/8/24	VER15-03	2015St1GC15	51.3620	104.5400	638	306	Kedr-1	88.1	11.7	0.1763	0.0151	0.0021	0.0155	0.0001	0.0000	7	-45.7	-26.1	-11.0	-275.5	-225.5	I+II
2015/8/24	VER15-03	2015St1GC15	51.3620	104.5400	638	325	Kedr-1	97.9	2.1	0.0016	0.0001	0.0000	0.0001	0.0000	0.0000	46	-47.7	-25.9	-10.2	-277.4	-220.4	I
2015/8/24	VER15-03	2015St1GC15	51.3620	104.5400	638	330	Kedr-1	96.6	3.4	0.0024	0.0001	0.0000	0.0003	0.0000	0.0000	29	-47.0	-25.7	n.d.	-275.4	-215.6	I
2015/9/5	VER15-03	2015St1GC18	51.3636	104.5407	617	254	Kedr-1	85.6	14.4	0.0096	0.0008	0.0001	0.0113	0.0000	0.0000	6	-46.6	-25.8	n.d.	-278.7	-215.9	II
2015/9/5	VER15-03	2015St1GC18	51.3636	104.5407	617	260	Kedr-1	86.6	13.4	0.0064	0.0003	0.0001	0.0033	0.0000	0.0000	6	-46.9	-25.8	n.d.	-278.2	-216.6	II
2015/9/5	VER15-03	2015St1GC18	51.3636	104.5407	617	263	Kedr-1	86.0	14.0	0.0118	0.0008	0.0001	0.0099	0.0000	0.0000	6	-47.1	-25.7	n.d.	-279.4	-221.1	II
2015/9/5	VER15-03	2015St1GC19	51.3623	104.5410	633	1	Kedr-1	97.7	2.3	0.0012	0.0000	0.0000	0.0002	0.0000	0.0000	43	-47.5	-25.7	n.d.	-274.7	-210.1	I
2015/9/5	VER15-03	2015St1GC19	51.3623	104.5410	633	20	Kedr-1	97.4	2.6	0.0020	0.0000	0.0000	0.0003	0.0000	0.0000	37	-47.6	-25.7	n.d.	-274.3	-211.1	I
2015/9/5	VER15-03	2015St1GC19	51.3623	104.5410	633	30	Kedr-1	97.1	2.9	0.0010	0.0000	0.0000	0.0001	0.0000	0.0000	33	-47.8	-25.3	n.d.	-275.4	-209.8	I
2016/8/26	VER16-03	2016St18GC1	51.3620	104.5359	636	60	Kedr-1	94.7	5.3	0.0198	0.0022	0.0006	0.0080	0.0000	0.0000	18	-45.8	-26.7	-8.5	-274.0	-227.4	I+II
2016/8/26	VER16-03	2016St18GC1	51.3620	104.5359	636	60	Kedr-1	94.0	6.0	0.0314	0.0030	0.0009	0.0099	0.0001	0.0000	16	-45.6	-26.9	-9.5	-274.1	-224.0	I+II
2016/8/26	VER16-03	2016St18GC1	51.3620	104.5359	636	70	Kedr-1	96.2	3.8	0.0039	0.0004	0.0001	0.0018	0.0000	0.0000	26	-46.0	-26.8	n.d.	-273.4	-222.8	I
2016/8/26	VER16-03	2016St18GC1	51.3620	104.5359	636	72	Kedr-1	96.6	3.4	0.0033	0.0003	0.0001	0.0003	0.0000	0.0000	28	-45.9	-27.0	n.d.	-274.1	-216.9	I
2016/8/26	VER16-03	2016St18GC1	51.3620	104.5359	636	80	Kedr-1	96.2	3.8	0.0049	0.0005	0.0001	0.0011	0.0001	0.0000	26	-46.2	-26.8	n.d.	-274.1	-219.0	I
2016/8/26	VER16-03	2016St18GC1	51.3620	104.5359	636	93	Kedr-1	97.2	2.8	0.0009	0.0000	0.0001	0.0000	0.0000	0.0000	35	-46.3	-26.7	n.d.	-274.2	-214.5	I
2016/8/26	VER16-03	2016St18GC2	51.3620	104.5359	634	35	Kedr-1	96.2	3.8	0.0055	0.0005	0.0001	0.0022	0.0000	0.0000	25	-46.8	-26.3	n.d.	-276.4	-207.0	I
2016/8/26	VER16-03	2016St18GC2	51.3620	104.5359	634	45	Kedr-1	97.0	3.0	0.0017	0.0001	0.0000	0.0001	0.0000	0.0000	32	-46.5	-26.7	n.d.	-272.8	-205.5	I
2016/8/26	VER16-03	2016St18GC2	51.3620	104.5359	634	122	Kedr-1	85.8	14.1	0.1592	0.0120	0.0020	0.0148	0.0003	0.0000	6	-46.0	-27.0	-10.5	-274.7	-215.4	II
2016/8/26	VER16-03	2016St18GC2	51.3620	104.5359	634	157	Kedr-1	85.5	14.2	0.2810	0.0114	0.0009	0.0239	0.0001	0.0000	6	-47.1	-26.9	-9.0	-275.5	-220.1	II
2016/8/26	VER16-03	2016St18GC2	51.3620	104.5359	634	258	Kedr-1	96.3	3.7	0.0023	0.0001	0.0001	0.0003	0.0000	0.0000	26	-46.5	-26.8	n.d.	-275.4	-208.7	I
2016/8/26	VER16-03	2016St18GC2	51.3620	104.5359	634	269	Kedr-1	97.9	2.1	0.0018	0.0000	0.0000	0.0003	0.0000	0.0000	47	-46.7	-26.9	n.d.	-273.0	-208.6	I
2016/8/26	VER16-03	2016St18GC2	51.3620	104.5359	634	277	Kedr-1	96.9	3.1	0.0009	0.0000	0.0000	0.0001	0.0000	0.0000	32	-46.5	-26.9	n.d.	-276.8	-206.0	I
2016/8/26	VER16-03	2016St18GC2	51.3620	104.5359	634	305	Kedr-1	96.0	4.0	0.0030	0.0002	0.0001	0.0010	0.0000	0.0000	24	-46.2	-26.8	n.d.	-276.4	-207.8	I
2016/8/26	VER16-03	2016St18GC2	51.3620	104.5359	634	333	Kedr-1	97.4	2.6	0.0006	0.0000	0.0001	0.0000	0.0000	0.0000	38	-46.6	-27.0	n.d.	-273.8	-207.5	I
2016/8/26	VER16-03	2016St18GC3	51.3636	104.5408	617	233	Kedr-1	86.0	14.0	0.0371	0.0031	0.0004	0.0167	0.0001	0.0000	6	-46.2	-26.7	n.d.	-278.0	-209.8	II
2016/8/26	VER16-03	2016St18GC3	51.3636	104.5408	617	242	Kedr-1	85.0	14.9	0.0718	0.0060	0.0008	0.0230	0.0002	0.0000	6	-46.3	-27.0	-10.1	-278.5	-209.7	II
2016/8/26	VER16-03	2016St18GC3	51.3636	104.5408	617	255	Kedr-1	84.8	15.1	0.1187	0.0099	0.0013	0.0305	0.0003	0.0000	6	-46.1	-27.0	-10.2	-280.5	-209.9	II
2016/8/26	VER16-03	2016St18GC3	51.3636	104.5408	617	262	Kedr-1	85.4	14.5	0.0583	0.0048	0.0007	0.0284	0.0001	0.0000	6	-46.1	-26.9	-9.8	-278.9	-208.4	II
2016/8/26	VER16-03	2016St18GC3	51.3636	104.5408	617	275	Kedr-1	84.4	15.5	0.0782	0.0065	0.0010	0.0323	0.0001	0.0000	5	-46.2	-26.8	-10.6	-278.8	-209.4	II
2016/8/26	VER16-03	2016St18GC3	51.3636	104.5408	617	287	Kedr-1	85.0	14.9	0.0747	0.0060	0.0010	0.0291	0.0001	0.0000	6	-46.2	-26.9	-10.3	-278.6	-209.4	II
2016/8/26	VER16-03	2016St18GC3	51.3636	104.5408	617	312	Kedr-1	85.2	14.6	0.1702	0.0145	0.0018	0.0350	0.0003	0.0000	6	-46.4	-27.0	-11.1	-279.2	-211.9	II
2016/8/27	VER16-03	2016St18GC4	51.3621	104.5347	625	159	Kedr-1	86.5	13.5	0.0118	0.0007	0.0002	0.0078	0.0000	0.0000	6	-46.3	-26.9	n.d.	-278.3	-210.3	II
2016/8/27	VER16-03	2016St18GC4	51.3621	104.5347	625	170	Kedr-1	86.9	13.1	0.0347	0.0023	0.0003	0.0146	0.0001	0.0000	7	-46.3	-26.8	n.d.	-276.7	-212.1	II
2016/8/27	VER16-03	2016St18GC4	51.3621	104.5347	625	174	Kedr-1	87.9	12.1	0.0197	0.0015	0.0003	0.0120	0.0000	0.0000	7	-46.4	-26.9	n.d.	-274.8	-209.7	I+II
2016/8/27	VER16-03	2016St18GC4	51.3621	104.5347	625	177	Kedr-1	90.6	9.4	0.0108	0.0007	0.0002	0.0067	0.0000	0.0000	10	-46.4	-26.9	n.d.	-275.8	-210.7	I+II

Table S1 (continue)

Date	Cruise Name	Core Name	Latitude [°N]	Longitude [°E]	Water Depth [m]	Sample Depth [cmblf]	Place	Molecular Composition: CH_4 [%]	C_2H_6 [%]	C_3H_8 [%]	i-C_4H_{10} [%]	n-C_4H_{10} [%]	neo-C_5H_{12} [%]	i-C_5H_{12} [%]	n-C_5H_{12} [%]	$C_1/(C_2+C_3)$	Isotopic Composition: CH_4 $\delta^{13}C$ [‰V-PDB]	C_2H_6 $\delta^{13}C$ [‰V-PDB]	C_3H_8 $\delta^{13}C$ [‰V-PDB]	CH_4 δ^2H [‰V-SMOW]	C_2H_6 δ^2H [‰V-SMOW]	Crystallographic Structure
2016/8/27	VER16-03	2016St18GC5	51.3621	104.5349	623	3	Kedr-1	95.9	4.1	0.0047	0.0005	0.0002	0.0018	0.0001	0.0000	23	-45.3	-26.8	n.d.	-276.3	-207.4	I
2016/8/27	VER16-03	2016St18GC5	51.3621	104.5349	623	20	Kedr-1	95.9	4.1	0.0033	0.0003	0.0002	0.0009	0.0000	0.0000	23	-45.4	-26.9	n.d.	-275.8	-209.1	I
2016/8/27	VER16-03	2016St18GC5	51.3621	104.5349	623	23	Kedr-1	96.0	3.9	0.0080	0.0007	0.0002	0.0023	0.0001	0.0000	24	-45.9	-26.8	n.d.	-276.1	-208.3	I
2016/8/27	VER16-03	2016St18GC6	51.3620	104.5350	617	205	Kedr-1	85.8	14.2	0.0167	0.0011	0.0003	0.0116	0.0000	0.0000	6	-46.1	-26.8	n.d.	-276.2	-211.6	II
2016/8/27	VER16-03	2016St18GC6	51.3620	104.5350	617	210	Kedr-1	85.9	14.0	0.0081	0.0005	0.0002	0.0083	0.0000	0.0000	6	-46.0	-26.6	n.d.	-277.2	-210.6	II
2016/8/27	VER16-03	2016St18GC6	51.3620	104.5350	617	230	Kedr-1	85.5	14.4	0.0980	0.0084	0.0013	0.0171	0.0001	0.0000	6	-46.1	-26.7	-12.3	-276.1	-210.9	II
2016/8/27	VER16-03	2016St18GC6	51.3620	104.5350	617	253	Kedr-1	86.0	13.9	0.0509	0.0043	0.0006	0.0163	0.0001	0.0000	6	-46.0	-26.8	-9.4	-276.1	-211.0	II
2016/8/27	VER16-03	2016St18GC6	51.3620	104.5350	617	272	Kedr-1	91.0	9.0	0.0481	0.0043	0.0007	0.0167	0.0001	0.0000	10	-45.9	-26.9	-10.3	-276.7	-211.3	I+II
2016/8/27	VER16-03	2016St18GC6	51.3620	104.5350	617	293	Kedr-1	96.1	3.9	0.0027	0.0001	0.0001	0.0004	0.0000	0.0000	25	-45.8	-27.1	n.d.	-275.8	-209.9	I
2016/8/28	VER16-03	2016St18GC7	51.3630	104.5409	617	149	Kedr-1	86.0	13.8	0.1024	0.0091	0.0011	0.0236	0.0002	0.0000	6	-46.5	-26.6	-10.8	-276.9	-212.1	II
2016/8/28	VER16-03	2016St18GC7	51.3630	104.5409	617	159	Kedr-1	96.6	3.4	0.0014	0.0000	0.0001	0.0002	0.0000	0.0000	28	-46.3	-27.0	n.d.	-276.5	-206.8	I
2016/8/28	VER16-03	2016St18GC7	51.3630	104.5409	617	173	Kedr-1	96.2	3.8	0.0003	0.0000	0.0001	0.0001	0.0000	0.0000	25	-46.1	-27.1	n.d.	-275.8	-207.5	I
2016/8/28	VER16-03	2016St18GC7	51.3630	104.5409	617	184	Kedr-1	95.1	4.9	0.0252	0.0028	0.0003	0.0069	0.0000	0.0000	19	-46.3	-26.8	-11.5	-277.1	-209.2	I
2016/8/28	VER16-03	2016St18GC7	51.3630	104.5409	617	194	Kedr-1	95.5	4.5	0.0052	0.0006	0.0002	0.0013	0.0000	0.0000	21	-46.9	-26.7	n.d.	-277.8	-207.7	I
2016/8/28	VER16-03	2016St18GC7	51.3630	104.5409	617	194	Kedr-1	95.3	4.7	0.0073	0.0008	0.0002	0.0022	0.0000	0.0000	20	-47.1	-26.9	n.d.	-276.9	-207.8	I
2016/8/26	VER16-03	2016St19GC1	51.3538	104.5445	588	149	Kedr-2	86.9	13.1	0.0088	0.0005	0.0002	0.0040	0.0000	0.0000	7	-44.7	-26.8	n.d.	-277.0	-208.7	II
2016/8/26	VER16-03	2016St19GC1	51.3538	104.5445	588	158	Kedr-2	87.1	12.9	0.0085	0.0003	0.0001	0.0039	0.0000	0.0000	7	-44.7	-27.0	n.d.	-276.3	-210.1	II
2016/8/26	VER16-03	2016St19GC1	51.3538	104.5445	588	162	Kedr-2	86.6	13.4	0.0106	0.0005	0.0002	0.0047	0.0000	0.0000	6	-44.7	-26.9	n.d.	-277.0	-208.5	II
2016/8/26	VER16-03	2016St19GC1	51.3538	104.5445	588	175	Kedr-2	86.9	13.1	0.0120	0.0006	0.0001	0.0069	0.0000	0.0000	7	-44.5	-27.0	n.d.	-276.6	-210.4	II
2016/8/26	VER16-03	2016St19GC1	51.3538	104.5445	588	177	Kedr-2	87.1	12.9	0.0120	0.0007	0.0001	0.0067	0.0000	0.0000	7	-44.4	-26.9	n.d.	-276.9	-209.6	II
2017/8/25	VER17-03	2017St12GC2	51.3621	104.5406	629	142	Kedr-1	95.7	4.3	0.0150	0.0010	0.0001	0.0077	0.0000	0.0000	22	-47.5	-27.2	n.d.	-275.1	-209.4	I
2017/8/25	VER17-03	2017St12GC2	51.3621	104.5406	629	160	Kedr-1	96.1	3.9	0.0055	0.0003	0.0001	0.0009	0.0000	0.0000	24	-47.1	-27.5	n.d.	-273.5	-209.7	I
2017/8/25	VER17-03	2017St12GC2	51.3621	104.5406	629	173	Kedr-1	95.8	4.2	0.0140	0.0009	0.0001	0.0048	0.0000	0.0000	23	-47.6	-27.2	n.d.	-274.8	-209.6	I
2017/8/25	VER17-03	2017St12GC2	51.3621	104.5406	629	184	Kedr-1	97.0	3.0	0.0009	0.0000	0.0000	0.0001	0.0000	0.0000	33	-47.6	-27.8	n.d.	-274.5	-211.0	I
2017/8/25	VER17-03	2017St12GC2	51.3621	104.5406	629	197	Kedr-1	96.6	3.4	0.0029	0.0001	0.0001	0.0002	0.0000	0.0000	28	-47.7	-27.4	n.d.	-273.6	-209.3	I
2017/8/25	VER17-03	2017St12GC2	51.3621	104.5406	629	198	Kedr-1	96.2	3.8	0.0042	0.0002	0.0000	0.0006	0.0000	0.0000	26	-47.4	-27.5	n.d.	-274.2	-208.9	I
2017/8/25	VER17-03	2017St12GC3	51.3630	104.5409	613	266	Kedr-1	85.2	14.8	0.0131	0.0008	0.0001	0.0121	0.0000	0.0000	6	-47.2	-27.5	n.d.	-275.3	-212.0	II
2017/8/25	VER17-03	2017St12GC3	51.3630	104.5409	613	293	Kedr-1	86.1	13.9	0.0120	0.0009	0.0002	0.0070	0.0000	0.0000	6	-47.1	-27.6	n.d.	-273.5	-212.4	II
2017/8/25	VER17-03	2017St13GC3	51.3536	104.5439	592	181	Kedr-2	87.1	12.9	0.0092	0.0005	0.0001	0.0059	0.0000	0.0000	7	-44.0	-27.6	n.d.	-275.0	-216.8	II
2017/8/25	VER17-03	2017St13GC3	51.3536	104.5439	592	216	Kedr-2	86.4	13.6	0.0264	0.0027	0.0002	0.0143	0.0000	0.0000	6	-44.0	-27.6	n.d.	-277.2	-211.6	II
2017/8/25	VER17-03	2017St13GC3	51.3536	104.5439	592	223	Kedr-2	85.9	14.1	0.0349	0.0030	0.0004	0.0151	0.0000	0.0000	6	-44.2	-27.6	n.d.	-278.1	-211.1	II
2017/8/25	VER17-03	2017St13GC3	51.3536	104.5439	592	232	Kedr-2	86.5	13.4	0.0200	0.0019	0.0001	0.0098	0.0000	0.0000	6	-44.1	-27.5	n.d.	-275.5	-211.8	II
2017/8/25	VER17-03	2017St13GC3	51.3536	104.5439	592	246	Kedr-2	95.0	5.0	0.0112	0.0008	0.0003	0.0019	0.0001	0.0000	19	-44.4	-27.7	n.d.	-274.9	-210.0	I
2017/8/25	VER17-03	2017St13GC3	51.3536	104.5439	592	253	Kedr-2	94.2	5.7	0.0297	0.0039	0.0002	0.0119	0.0000	0.0000	16	-44.5	-27.5	-11.6	-277.4	-208.9	I+II
2017/8/25	VER17-03	2017St13GC3	51.3536	104.5439	592	255	Kedr-2	95.8	4.2	0.0035	0.0002	0.0001	0.0007	0.0000	0.0000	23	-44.6	-27.4	n.d.	-276.4	-208.8	I

Table S2

								Molecular Composition					Isotopic Composition				
Date	Cruise Name	Core Name	Latitude [°N]	Longitude [°E]	Water Depth [m]	Sample Depth [cmblf]	Place	CH_4 [mM/L]	C_2H_6 [uM/L]	C_3H_8 [uM/L]	CO_2 [mM/L]	$C_1/(C_2+C_3)$	CH_4 $\delta^{13}C$ [‰V-PDB]	C_2H_6 $\delta^{13}C$ [‰V-PDB]	CO_2 $\delta^{13}C$ [‰V-PDB]	CH_4 δ^2H [‰V-SMOW]	C_2H_6 δ^2H [‰V-SMOW]
2015/8/16	VER15-03	2015St1GC1	51.3624	104.5354	620	20	Kedr-1	0.00	0.1	0.002	0.44	19	n.d.	n.d.	-22.5	n.d.	n.d.
2015/8/16	VER15-03	2015St1GC1	51.3624	104.5354	620	60	Kedr-1	0.48	6.1	0.003	0.83	79	-57.4	n.d.	-21.6	-265.4	-203.9
2015/8/16	VER15-03	2015St1GC1	51.3624	104.5354	620	100	Kedr-1	4.09	85.7	0.019	1.40	48	-51.6	n.d.	-16.0	-275.4	-214.3
2015/8/16	VER15-03	2015St1GC1	51.3624	104.5354	620	140	Kedr-1	7.92	185.6	0.025	1.27	43	-48.4	n.d.	-7.9	-274.8	-222.1
2015/8/16	VER15-03	2015St1GC1	51.3624	104.5354	620	180	Kedr-1	3.63	111.6	0.016	1.53	32	-46.1	n.d.	-4.1	-271.5	-223.2
2015/8/16	VER15-03	2015St1GC1	51.3624	104.5354	620	220	Kedr-1	4.17	128.2	0.029	1.91	33	-45.3	n.d.	-2.1	-269.8	-225.1
2015/8/24	VER15-03	2015St1GC3	51.3622	104.5332	604	10	Kedr-1	10.72	187.3	0.020	0.64	57	-47.8	-25.8	-2.6	-277.5	-226.5
2015/8/16	VER15-03	2015St1GC4	51.3625	104.5345	642	10	Kedr-1	0.21	2.5	0.003	1.25	83	-63.1	-25.2	-21.4	n.d.	n.d.
2015/8/16	VER15-03	2015St1GC4	51.3625	104.5345	642	50	Kedr-1	2.83	78.6	0.009	0.95	36	-54.1	n.d.	-17.9	-282.8	-207.1
2015/8/16	VER15-03	2015St1GC4	51.3625	104.5345	642	90	Kedr-1	2.19	66.2	0.006	0.68	33	-49.9	n.d.	-16.0	-290.7	-208.3
2015/8/16	VER15-03	2015St1GC4	51.3625	104.5345	642	130	Kedr-1	24.38	724.7	0.049	1.50	34	-49.9	n.d.	-12.4	-289.5	-208.7
2015/8/16	VER15-03	2015St1GC4	51.3625	104.5345	642	170	Kedr-1	11.87	404.6	0.028	1.50	29	-48.4	n.d.	-9.5	-286.4	-209.6
2015/8/16	VER15-03	2015St1GC4	51.3625	104.5345	642	210	Kedr-1	8.74	313.3	0.022	1.39	28	-48.1	n.d.	-6.8	-283.6	-209.6
2015/8/16	VER15-03	2015St1GC4	51.3625	104.5345	642	250	Kedr-1	21.88	770.5	0.054	1.41	28	-47.1	n.d.	-5.0	-281.9	-209.9
2015/8/16	VER15-03	2015St1GC6	51.3557	104.5411	689	20	Kedr-1	0.03	0.0	0.004	0.66	757	-97.4	n.d.	-21.3	n.d.	n.d.
2015/8/16	VER15-03	2015St1GC6	51.3557	104.5411	689	60	Kedr-1	0.14	0.3	0.001	0.47	468	-91.0	-27.5	-13.9	n.d.	n.d.
2015/8/16	VER15-03	2015St1GC6	51.3557	104.5411	689	100	Kedr-1	0.21	0.6	0.002	0.35	343	-88.7	-25.9	-13.7	n.d.	n.d.
2015/8/16	VER15-03	2015St1GC6	51.3557	104.5411	689	140	Kedr-1	0.70	2.8	0.004	0.67	255	-87.1	-26.3	-12.4	-309.7	-281.0
2015/8/16	VER15-03	2015St1GC6	51.3557	104.5411	689	180	Kedr-1	0.65	2.9	0.003	0.68	226	-86.7	-27.0	-11.9	-308.1	-270.2
2015/8/16	VER15-03	2015St1GC6	51.3557	104.5411	689	220	Kedr-1	0.59	3.1	0.002	0.30	189	-86.4	-27.2	-13.8	-305.7	-261.3
2015/8/25	VER15-03	2015St1GC7	51.3539	104.5438	591	20	Kedr-2	0.00	0.0	0.001	0.11	554	n.d.	n.d.	-17.1	n.d.	n.d.
2015/8/25	VER15-03	2015St1GC7	51.3539	104.5438	591	60	Kedr-2	0.10	0.5	0.002	0.45	206	-54.7	-25.7	-14.9	n.d.	n.d.
2015/8/25	VER15-03	2015St1GC7	51.3539	104.5438	591	100	Kedr-2	1.76	23.8	0.007	0.32	74	-48.6	-26.2	-12.0	-267.3	-225.2
2015/8/25	VER15-03	2015St1GC7	51.3539	104.5438	591	140	Kedr-2	4.94	70.1	0.014	0.78	71	-47.2	-26.7	-8.0	-273.3	-212.8
2015/8/25	VER15-03	2015St1GC7	51.3539	104.5438	591	180	Kedr-2	6.49	103.7	0.018	0.36	63	-45.5	-26.7	0.8	-273.3	-225.5
2015/8/25	VER15-03	2015St1GC7	51.3539	104.5438	591	220	Kedr-2	21.11	351.7	0.046	0.83	60	-44.5	-26.4	7.3	-272.5	-230.3
2015/8/25	VER15-03	2015St1GC7	51.3539	104.5438	591	260	Kedr-2	14.41	260.9	0.035	0.72	55	-43.9	-26.2	9.1	-273.6	-228.9
2015/8/25	VER15-03	2015St1GC7	51.3539	104.5438	591	290	Kedr-2	12.28	237.4	0.038	1.96	52	-43.4	-26.1	20.1	-270.9	-234.0
2015/8/16	VER15-03	2015St1GC8	51.3623	104.5410	625	30	Kedr-1	5.04	115.7	0.137	0.68	44	-49.2	-26.4	0.6	-273.5	-208.8
2015/8/16	VER15-03	2015St1GC8	51.3623	104.5410	625	70	Kedr-1	1.89	73.9	0.150	0.82	25	-46.4	-26.7	4.4	-270.6	-209.6
2015/8/16	VER15-03	2015St1GC8	51.3623	104.5410	625	110	Kedr-1	3.13	294.0	0.920	1.70	11	-45.3	-26.5	8.9	-270.4	-214.3
2015/8/16	VER15-03	2015St1GC9	51.3631	104.5409	616	20	Kedr-1	0.01	0.0	0.002	0.25	252	-48.4	n.d.	-18.9	n.d.	n.d.
2015/8/16	VER15-03	2015St1GC9	51.3631	104.5409	616	60	Kedr-1	2.01	31.7	0.021	0.78	63	-56.6	-26.5	-10.6	-280.1	-197.5
2015/8/16	VER15-03	2015St1GC9	51.3631	104.5409	616	100	Kedr-1	5.60	115.3	0.099	0.71	49	-50.9	-26.3	-3.4	-280.4	-210.0
2015/8/16	VER15-03	2015St1GC9	51.3631	104.5409	616	140	Kedr-1	8.59	221.1	0.190	0.79	39	-48.1	-25.9	3.1	-276.7	-209.8
2015/8/16	VER15-03	2015St1GC9	51.3631	104.5409	616	180	Kedr-1	3.17	115.9	0.102	1.32	27	-46.5	-25.9	9.1	-274.2	-210.2
2015/8/16	VER15-03	2015St1GC9	51.3631	104.5409	616	220	Kedr-1	4.60	140.2	0.047	1.83	33	-46.2	-25.8	13.2	-274.4	-208.6
2015/8/16	VER15-03	2015St1GC10	51.3616	104.5405	609	20	Kedr-1	3.02	144.2	0.125	1.26	21	-44.3	-26.3	-6.7	-269.3	-211.2
2015/8/16	VER15-03	2015St1GC11	51.3620	104.5359	636	20	Kedr-1	15.35	419.7	1.958	1.42	36	-52.0	-25.8	-4.4	-283.2	-213.3
2015/8/16	VER15-03	2015St1GC11	51.3620	104.5359	636	60	Kedr-1	7.04	754.6	5.240	1.44	9	-47.9	-25.8	0.8	-278.6	-223.7
2015/8/16	VER15-03	2015St1GC11	51.3620	104.5359	636	100	Kedr-1	8.50	363.5	4.298	2.21	23	-46.3	-25.9	3.2	-271.5	-215.6
2015/8/24	VER15-03	2015St1GC12	51.3613	104.5355	604	30	Kedr-1	11.63	211.2	0.027	1.26	55	-48.4	-26.1	6.7	-273.5	-242.7
2015/8/24	VER15-03	2015St1GC12	51.3613	104.5355	604	40	Kedr-1	6.87	186.3	0.046	1.94	37	-45.1	-25.7	15.7	-269.9	-243.7
2015/8/24	VER15-03	2015St1GC13	51.3622	104.5333	604	10	Kedr-1	13.21	626.6	0.242	0.69	21	-44.3	-26.0	1.6	-276.1	-220.9
2015/8/24	VER15-03	2015St1GC14	51.3621	104.5347	625	20	Kedr-1	0.46	5.8	0.005	0.56	80	-59.3	-25.8	-10.3	-279.6	-256.8
2015/8/24	VER15-03	2015St1GC14	51.3621	104.5347	625	60	Kedr-1	15.92	326.7	0.036	0.61	49	-46.9	-26.0	-4.0	-272.5	-241.6

Table S2 (continue)

Date	Cruise Name	Core Name	Latitude [°N]	Longitude [°E]	Water Depth [m]	Sample Depth [cmblf]	Place	Molecular Composition					Isotopic Composition				
								CH_4 [mM/L]	C_2H_6 [uM/L]	C_3H_8 [uM/L]	CO_2 [mM/L]	$C_1/(C_2+C_3)$	CH_4 $\delta^{13}C$ [‰V-PDB]	C_2H_6 $\delta^{13}C$ [‰V-PDB]	CO_2 $\delta^{13}C$ [‰V-PDB]	CH_4 δ^2H [‰V-SMOW]	C_2H_6 δ^2H [‰V-SMOW]
2015/8/24	VER15-03	2015St1GC14	51.3621	104.5347	625	100	Kedr-1	7.03	204.0	0.029	0.79	34	-45.0	-25.6	7.8	-270.4	-228.5
2015/8/24	VER15-03	2015St1GC14	51.3621	104.5347	625	140	Kedr-1	5.17	174.9	0.036	0.87	30	-44.2	-26.1	6.9	-267.9	-230.7
2015/8/24	VER15-03	2015St1GC14	51.3621	104.5347	625	162	Kedr-1	4.49	332.4	0.146	2.59	14	-44.1	-26.2	17.8	-270.6	-238.5
2015/8/24	VER15-03	2015St1GC15	51.3620	104.5400	638	20	Kedr-1	0.47	18.7	0.036	0.48	25	-62.8	-25.9	-11.5	-277.9	-246.9
2015/8/24	VER15-03	2015St1GC15	51.3620	104.5400	638	60	Kedr-1	4.84	309.4	0.735	1.30	16	-59.5	-25.5	1.5	-297.1	-220.1
2015/8/24	VER15-03	2015St1GC15	51.3620	104.5400	638	100	Kedr-1	4.00	232.8	0.457	1.46	17	-55.9	-25.2	-0.2	-292.7	-224.3
2015/8/24	VER15-03	2015St1GC15	51.3620	104.5400	638	140	Kedr-1	2.66	125.7	0.193	1.69	21	-52.8	-25.4	-1.6	-286.6	-224.0
2015/8/24	VER15-03	2015St1GC15	51.3620	104.5400	638	180	Kedr-1	2.41	87.9	0.089	2.35	27	-50.1	-25.6	-3.1	-282.0	-224.4
2015/8/24	VER15-03	2015St1GC15	51.3620	104.5400	638	220	Kedr-1	1.71	59.6	0.089	1.54	29	-48.5	-26.2	-3.5	-278.6	-228.2
2015/8/24	VER15-03	2015St1GC15	51.3620	104.5400	638	260	Kedr-1	3.32	247.7	0.226	2.36	13	-47.5	-26.4	1.6	-277.3	-234.3
2015/8/24	VER15-03	2015St1GC15	51.3620	104.5400	638	300	Kedr-1	3.11	184.7	0.587	2.34	17	-46.9	-26.4	6.9	-275.6	-230.1
2015/8/24	VER15-03	2015St1GC15	51.3620	104.5400	638	315	Kedr-1	3.17	69.6	0.079	2.23	45	-47.4	-26.4	7.0	-274.6	-230.6
2015/8/25	VER15-03	2015St1GC16	51.3549	104.5435	616	20	Kedr-2	0.01	0.0	0.001	0.44	1455	-47.0	n.d.	-12.8	n.d.	n.d.
2015/8/25	VER15-03	2015St1GC16	51.3549	104.5435	616	60	Kedr-2	0.06	0.5	0.001	0.91	141	-51.4	-26.1	-11.6	n.d.	n.d.
2015/8/25	VER15-03	2015St1GC16	51.3549	104.5435	616	100	Kedr-2	0.98	10.2	0.005	0.31	97	-52.6	-25.4	-14.9	-271.2	-226.3
2015/8/25	VER15-03	2015St1GC16	51.3549	104.5435	616	140	Kedr-2	5.09	59.7	0.014	0.33	85	-52.9	-25.9	-13.2	-279.8	-234.9
2015/8/25	VER15-03	2015St1GC16	51.3549	104.5435	616	180	Kedr-2	5.99	79.7	0.015	0.74	75	-52.4	-26.5	-3.1	-286.0	-212.7
2015/8/25	VER15-03	2015St1GC16	51.3549	104.5435	616	220	Kedr-2	10.37	140.6	0.021	0.16	74	-52.7	-26.6	-1.9	-286.7	-218.3
2015/8/25	VER15-03	2015St1GC16	51.3549	104.5435	616	250	Kedr-2	12.15	171.3	0.025	0.64	71	-52.0	-26.5	5.5	-282.6	-220.9
2015/8/25	VER15-03	2015St1GC17	51.3603	104.5432	628	20	Kedr-1	0.01	0.0	0.003	0.29	1224	-53.3	n.d.	-15.2	n.d.	n.d.
2015/8/25	VER15-03	2015St1GC17	51.3603	104.5432	628	60	Kedr-1	0.20	1.3	0.004	0.48	156	-66.3	-25.3	-11.7	n.d.	n.d.
2015/8/25	VER15-03	2015St1GC17	51.3603	104.5432	628	100	Kedr-1	1.00	8.5	0.005	0.66	117	-62.8	-25.3	-8.4	-291.7	n.d.
2015/8/25	VER15-03	2015St1GC17	51.3603	104.5432	628	140	Kedr-1	3.56	38.9	0.011	0.29	91	-60.0	-26.2	-9.3	-289.3	-232.0
2015/8/25	VER15-03	2015St1GC17	51.3603	104.5432	628	180	Kedr-1	6.18	71.6	0.016	0.31	86	-59.2	-26.6	-4.7	-292.4	-211.4
2015/8/25	VER15-03	2015St1GC17	51.3603	104.5432	628	220	Kedr-1	6.23	74.6	0.020	0.16	83	-58.8	-26.8	-5.3	-290.4	-226.0
2015/8/25	VER15-03	2015St1GC17	51.3603	104.5432	628	260	Kedr-1	6.10	77.6	0.018	0.57	79	-58.0	-26.7	0.7	-290.7	-216.1
2016/8/26	VER16-03	2016St18GC1	51.3620	104.5359	636	10	Kedr-1	5.49	73.1	0.045	0.34	75	-54.6	-27.0	1.3	-281.1	-208.7
2016/8/26	VER16-03	2016St18GC1	51.3620	104.5359	636	30	Kedr-1	13.67	222.9	0.172	0.86	61	-50.1	-26.5	8.5	-277.4	-211.8
2016/8/26	VER16-03	2016St18GC1	51.3620	104.5359	636	40	Kedr-1	8.83	195.8	0.209	1.13	45	-47.1	-26.0	10.1	-275.7	-212.3
2016/8/26	VER16-03	2016St18GC1	51.3620	104.5359	636	50	Kedr-1	12.57	1608.3	1.974	0.74	8	-46.4	-26.3	10.7	-276.8	-221.0
2016/8/26	VER16-03	2016St18GC1	51.3620	104.5359	636	70	Kedr-1	5.84	268.8	0.022	0.56	22	-45.8	-25.8	14.2	-277.3	-232.0
2016/8/26	VER16-03	2016St18GC2	51.3620	104.5359	634	20	Kedr-1	7.55	170.7	0.072	0.57	44	-47.8	-25.7	0.1	-275.3	-212.7
2016/8/26	VER16-03	2016St18GC2	51.3620	104.5359	634	60	Kedr-1	3.76	162.8	0.146	1.18	23	-47.3	-25.8	8.6	-279.0	-210.5
2016/8/26	VER16-03	2016St18GC2	51.3620	104.5359	634	90	Kedr-1	3.17	98.3	0.088	0.81	32	-48.0	-26.2	7.2	-279.5	-210.9
2016/8/26	VER16-03	2016St18GC2	51.3620	104.5359	634	130	Kedr-1	1.49	114.8	0.334	1.13	13	-45.7	-26.3	7.6	-276.4	-213.7
2016/8/26	VER16-03	2016St18GC2	51.3620	104.5359	634	190	Kedr-1	1.22	52.6	0.111	0.64	23	-45.6	-26.8	5.3	-269.6	-209.0
2016/8/26	VER16-03	2016St18GC2	51.3620	104.5359	634	210	Kedr-1	3.99	160.8	0.171	1.77	25	-45.3	-26.1	8.9	-274.3	-212.3
2016/8/26	VER16-03	2016St18GC2	51.3620	104.5359	634	230	Kedr-1	2.12	48.9	0.118	2.15	43	-44.8	-26.2	10.2	-271.3	-208.4
2016/8/26	VER16-03	2016St18GC2	51.3620	104.5359	634	263	Kedr-1	3.50	175.1	0.170	0.98	20	-45.3	-26.2	14.9	-277.0	-212.6
2016/8/26	VER16-03	2016St18GC2	51.3620	104.5359	634	320	Kedr-1	2.36	67.9	0.087	1.63	35	-43.4	-26.1	19.8	-267.8	-204.2
2016/8/26	VER16-03	2016St18GC3	51.3636	104.5408	617	20	Kedr-1	0.13	31.1	0.087	0.19	4	-47.7	-27.3	-20.1	n.d.	-210.9
2016/8/26	VER16-03	2016St18GC3	51.3636	104.5408	617	60	Kedr-1	2.86	147.9	0.260	0.52	19	-53.5	-26.5	-12.2	-273.4	-211.5
2016/8/26	VER16-03	2016St18GC3	51.3636	104.5408	617	100	Kedr-1	13.74	451.2	0.075	0.49	30	-50.0	-26.0	-8.1	-278.9	-210.6
2016/8/26	VER16-03	2016St18GC3	51.3636	104.5408	617	140	Kedr-1	9.02	405.7	0.046	0.88	22	-46.4	-26.0	7.9	-275.8	-210.8
2016/8/26	VER16-03	2016St18GC3	51.3636	104.5408	617	180	Kedr-1	4.16	249.8	0.038	0.63	17	-45.0	-25.3	11.1	-271.7	-211.0
2016/8/26	VER16-03	2016St18GC3	51.3636	104.5408	617	220	Kedr-1	2.93	201.2	0.153	0.70	15	-43.0	-25.3	15.9	-268.9	-211.8

Table S2 (continue)

								Molecular Composition					Isotopic Composition				
Date	Cruise Name	Core Name	Latitude [°N]	Longitude [°E]	Water Depth [m]	Sample Depth [cmblf]	Place	CH_4 [mM/L]	C_2H_6 [uM/L]	C_3H_8 [uM/L]	CO_2 [mM/L]	$C_1/(C_2+C_3)$	CH_4 $\delta^{13}C$ [‰V-PDB]	C_2H_6 $\delta^{13}C$ [‰V-PDB]	CO_2 $\delta^{13}C$ [‰V-PDB]	CH_4 δ^2H [‰V-SMOW]	C_2H_6 δ^2H [‰V-SMOW]
2016/8/26	VER16-03	2016St18GC3	51.3636	104.5408	617	270	Kedr-1	6.29	584.6	1.057	0.83	11	-44.6	-25.8	19.4	-274.8	-215.2
2016/8/26	VER16-03	2016St18GC3	51.3636	104.5408	617	295	Kedr-1	3.54	236.0	0.282	0.67	15	-44.0	-25.9	19.6	-268.8	-211.2
2016/8/26	VER16-03	2016St18GC3	51.3636	104.5408	617	320	Kedr-1	1.28	52.9	0.060	0.40	24	-44.2	-26.2	21.0	-271.9	-209.7
2016/8/27	VER16-03	2016St18GC4	51.3621	104.5347	625	20	Kedr-1	0.56	83.1	0.103	0.18	7	-48.3	-26.9	-11.2	-265.9	-216.2
2016/8/27	VER16-03	2016St18GC4	51.3621	104.5347	625	60	Kedr-1	6.60	105.0	0.019	0.51	63	-48.4	-26.0	-4.4	-276.3	-205.9
2016/8/27	VER16-03	2016St18GC4	51.3621	104.5347	625	100	Kedr-1	2.87	131.4	0.079	0.44	22	-44.9	-26.4	9.7	-273.1	-209.9
2016/8/27	VER16-03	2016St18GC4	51.3621	104.5347	625	140	Kedr-1	2.98	88.6	0.024	0.30	34	-44.6	-25.8	16.9	-270.0	-208.6
2016/8/27	VER16-03	2016St18GC4	51.3621	104.5347	625	150	Kedr-1	6.92	219.0	0.069	0.67	32	-44.8	-26.0	15.6	-273.8	-210.4
2016/8/27	VER16-03	2016St18GC6	51.3620	104.5350	617	30	Kedr-1	0.10	1.4	0.009	0.29	71	-56.4	-26.2	-14.7	n.d.	n.d.
2016/8/27	VER16-03	2016St18GC6	51.3620	104.5350	617	70	Kedr-1	2.90	68.1	0.011	0.07	43	-53.2	-26.3	-11.2	-278.5	-206.0
2016/8/27	VER16-03	2016St18GC6	51.3620	104.5350	617	110	Kedr-1	11.31	320.4	0.030	0.70	35	-47.3	-25.9	8.4	-277.3	-210.6
2016/8/27	VER16-03	2016St18GC6	51.3620	104.5350	617	150	Kedr-1	7.35	308.4	0.040	0.48	24	-45.1	-25.6	13.4	-273.2	-210.3
2016/8/27	VER16-03	2016St18GC6	51.3620	104.5350	617	190	Kedr-1	4.44	299.8	0.191	0.68	15	-44.4	-26.1	18.8	-275.1	-213.9
2016/8/27	VER16-03	2016St18GC6	51.3620	104.5350	617	247	Kedr-1	3.36	225.4	0.087	0.43	15	-44.2	-26.2	19.4	-273.2	-210.7
2016/8/27	VER16-03	2016St18GC6	51.3620	104.5350	617	265	Kedr-1	2.61	333.2	0.105	0.23	8	-44.7	-25.9	18.6	-279.6	-210.6
2016/8/28	VER16-03	2016St18GC7	51.3630	104.5409	617	30	Kedr-1	2.39	70.9	0.020	0.32	34	-55.6	-26.4	-6.9	-275.1	-209.0
2016/8/28	VER16-03	2016St18GC7	51.3630	104.5409	617	70	Kedr-1	15.12	577.1	0.057	0.44	26	-47.9	-25.5	4.1	-277.4	-210.3
2016/8/28	VER16-03	2016St18GC7	51.3630	104.5409	617	110	Kedr-1	7.32	430.7	0.102	0.56	17	-44.7	-25.0	9.7	-272.8	-208.8
2016/8/28	VER16-03	2016St18GC7	51.3630	104.5409	617	140	Kedr-1	6.74	366.4	0.723	0.78	18	-45.4	-25.9	19.4	-272.9	-209.8
2016/8/28	VER16-03	2016St18GC7	51.3630	104.5409	617	168	Kedr-1	4.38	196.1	0.089	0.59	22	-44.7	-26.1	17.2	-274.0	-209.3
2016/8/28	VER16-03	2016St18GC7	51.3630	104.5409	617	196	Kedr-1	6.48	362.8	0.194	1.01	18	-44.7	-25.7	15.2	-276.2	-209.2
2016/8/26	VER16-03	2016St19GC1	51.3538	104.5445	588	20	Kedr-2	4.64	73.5	0.020	0.51	63	-46.5	-26.4	-3.8	-270.3	-206.4
2016/8/26	VER16-03	2016St19GC1	51.3538	104.5445	588	60	Kedr-2	13.16	220.5	0.029	0.95	60	-44.4	-25.8	14.1	-273.2	-201.7
2016/8/26	VER16-03	2016St19GC1	51.3538	104.5445	588	100	Kedr-2	13.23	243.8	0.032	1.00	54	-43.3	-25.6	23.0	-269.8	-203.5
2016/8/26	VER16-03	2016St19GC1	51.3538	104.5445	588	140	Kedr-2	6.31	211.7	0.293	1.54	30	-42.3	-25.6	23.6	-269.5	-203.8
2016/8/26	VER16-03	2016St19GC1	51.3538	104.5445	588	153	Kedr-2	8.40	356.2	0.182	2.85	24	-42.2	-26.0	28.1	-269.2	-207.3
2016/8/27	VER16-03	2016St19GC2	51.3539	104.5445	588	30	Kedr-2	0.00	0.0	0.006	0.14	156	n.d.	n.d.	-13.8	n.d.	n.d.
2016/8/27	VER16-03	2016St19GC2	51.3539	104.5445	588	70	Kedr-2	0.27	2.5	0.010	0.28	110	-53.7	-23.4	-15.4	n.d.	n.d.
2016/8/27	VER16-03	2016St19GC2	51.3539	104.5445	588	110	Kedr-2	2.56	44.3	0.012	0.30	58	-48.0	-25.3	-9.0	-274.1	-206.7
2016/8/27	VER16-03	2016St19GC2	51.3539	104.5445	588	150	Kedr-2	11.02	228.6	0.030	0.46	48	-45.8	-25.6	2.7	-276.5	-205.8
2016/8/27	VER16-03	2016St19GC2	51.3539	104.5445	588	190	Kedr-2	15.06	361.3	0.046	0.72	42	-44.9	-25.2	15.1	-276.1	-208.5
2016/8/27	VER16-03	2016St19GC2	51.3539	104.5445	588	230	Kedr-2	15.29	391.0	0.047	0.70	39	-44.2	-25.1	19.1	-275.8	-205.8
2016/8/27	VER16-03	2016St19GC2	51.3539	104.5445	588	270	Kedr-2	5.06	160.4	0.024	1.40	32	-43.1	-25.3	22.5	-275.5	-202.5
2016/8/27	VER16-03	2016St19GC2	51.3539	104.5445	588	310	Kedr-2	5.03	171.8	0.026	1.10	29	-42.6	-25.2	18.7	-273.8	-203.9
2016/8/27	VER16-03	2016St19GC2	51.3539	104.5445	588	340	Kedr-2	5.02	175.3	0.029	1.29	29	-42.4	-25.2	25.0	-274.7	-208.8
2016/8/27	VER16-03	2016St19GC3	51.3525	104.5441	614	20	Kedr-2	0.01	0.0	0.009	0.32	233	-46.6	n.d.	-12.5	n.d.	n.d.
2016/8/27	VER16-03	2016St19GC3	51.3525	104.5441	614	60	Kedr-2	0.97	8.8	0.009	0.32	110	-53.3	-25.0	-10.8	-271.9	-205.4
2016/8/27	VER16-03	2016St19GC3	51.3525	104.5441	614	100	Kedr-2	10.31	118.8	0.024	0.71	87	-50.2	-25.8	-2.4	-282.3	-200.3
2016/8/27	VER16-03	2016St19GC3	51.3525	104.5441	614	130	Kedr-2	14.14	171.6	0.051	0.59	82	-49.5	-25.6	5.0	-282.9	-199.0
2016/8/27	VER16-03	2016St19GC4	51.3537	104.5427	596	20	Kedr-2	0.00	0.0	0.009	0.23	83	n.d.	n.d.	-16.4	n.d.	n.d.
2016/8/27	VER16-03	2016St19GC4	51.3537	104.5427	596	60	Kedr-2	0.14	1.1	0.009	0.18	124	-52.8	-24.3	-16.6	n.d.	n.d.
2016/8/27	VER16-03	2016St19GC4	51.3537	104.5427	596	100	Kedr-2	0.95	16.4	0.008	0.23	58	-49.4	-25.4	-15.5	-270.9	-214.3
2016/8/27	VER16-03	2016St19GC4	51.3537	104.5427	596	140	Kedr-2	4.54	94.4	0.018	0.18	48	-46.9	-26.0	-4.7	-276.0	-206.2
2016/8/27	VER16-03	2016St19GC4	51.3537	104.5427	596	180	Kedr-2	9.46	218.6	0.029	0.53	43	-44.9	-25.8	8.5	-276.9	-211.4
2016/8/27	VER16-03	2016St19GC4	51.3537	104.5427	596	220	Kedr-2	10.83	266.5	0.033	0.58	41	-43.8	-25.6	15.8	-271.8	-210.5
2016/8/27	VER16-03	2016St19GC5	51.3539	104.5446	588	30	Kedr-2	0.00	0.0	0.008	0.21	70	n.d.	n.d.	-12.1	n.d.	n.d.

Table S2 (continue)

Date	Cruise Name	Core Name	Latitude [°N]	Longitude [°E]	Water Depth [m]	Sample Depth [cmblf]	Place	Molecular Composition CH_4 [mM/L]	C_2H_6 [uM/L]	C_3H_8 [uM/L]	CO_2 [mM/L]	$C_1/(C_2+C_3)$	Isotopic Composition CH_4 $\delta^{13}C$ [‰V-PDB]	C_2H_6 $\delta^{13}C$ [‰V-PDB]	CO_2 $\delta^{13}C$ [‰V-PDB]	CH_4 δ^2H [‰V-SMOW]	C_2H_6 δ^2H [‰V-SMOW]
2016/8/27	VER16-03	2016St19GC5	51.3539	104.5446	588	70	Kedr-2	0.14	1.2	0.006	0.18	111	-53.2	-23.9	-12.3	n.d.	n.d.
2016/8/27	VER16-03	2016St19GC5	51.3539	104.5446	588	110	Kedr-2	1.40	23.4	0.010	0.25	60	-48.0	-25.0	-8.7	-266.5	-205.0
2016/8/27	VER16-03	2016St19GC5	51.3539	104.5446	588	150	Kedr-2	7.62	160.5	0.029	0.40	47	-46.0	-25.6	-2.5	-274.6	-202.7
2016/8/27	VER16-03	2016St19GC5	51.3539	104.5446	588	190	Kedr-2	12.78	298.1	0.040	0.84	43	-45.0	-25.5	13.4	-274.9	-208.6
2016/8/27	VER16-03	2016St19GC5	51.3539	104.5446	588	230	Kedr-2	10.78	270.6	0.036	0.86	40	-44.3	-25.4	20.1	-273.6	-208.8
2016/8/27	VER16-03	2016St19GC5	51.3539	104.5446	588	310	Kedr-2	3.01	102.9	0.019	1.27	29	-43.1	-25.4	23.2	-270.0	-207.6
2016/8/27	VER16-03	2016St19GC5	51.3539	104.5446	588	350	Kedr-2	7.09	221.6	0.031	0.76	32	-42.7	-25.3	20.9	-271.2	-208.9
2016/8/27	VER16-03	2016St21GC1	51.3551	104.5530	779	20	Kedr-2	0.04	0.0	0.007	0.26	1066	-88.6	n.d.	-16.2	n.d.	n.d.
2016/8/27	VER16-03	2016St21GC1	51.3551	104.5530	779	60	Kedr-2	0.28	0.4	0.010	0.13	700	-93.3	-31.5	-18.6	n.d.	n.d.
2016/8/27	VER16-03	2016St21GC1	51.3551	104.5530	779	100	Kedr-2	0.42	0.6	0.007	0.15	661	-89.9	-31.0	-11.9	n.d.	n.d.
2016/8/27	VER16-03	2016St21GC1	51.3551	104.5530	779	140	Kedr-2	0.64	1.1	0.010	0.14	578	-86.7	-31.2	-13.4	-311.0	n.d.
2016/8/27	VER16-03	2016St21GC1	51.3551	104.5530	779	180	Kedr-2	1.76	3.2	0.013	0.32	543	-84.6	-31.8	-12.7	-305.0	n.d.
2016/8/27	VER16-03	2016St21GC1	51.3551	104.5530	779	220	Kedr-2	1.51	2.8	0.009	0.10	534	-83.4	-30.9	-17.7	-303.6	n.d.
2016/8/27	VER16-03	2016St22GC1	51.3527	104.5410	704	20	Kedr-2	0.05	0.0	0.007	0.34	821	-94.1	n.d.	-14.6	n.d.	n.d.
2016/8/27	VER16-03	2016St22GC1	51.3527	104.5410	704	60	Kedr-2	0.22	0.2	0.008	0.17	859	-91.0	-30.0	-10.4	n.d.	n.d.
2016/8/27	VER16-03	2016St22GC1	51.3527	104.5410	704	100	Kedr-2	0.97	1.3	0.018	0.26	730	-85.3	-30.5	-10.1	-311.6	n.d.
2016/8/27	VER16-03	2016St22GC1	51.3527	104.5410	704	140	Kedr-2	0.92	1.4	0.012	0.24	665	-81.8	-30.5	-8.3	-303.0	n.d.
2016/8/28	VER16-03	2016St25GC1	51.3645	104.5459	729	20	Kedr-1	0.13	1.0	0.007	0.26	129	-80.6	-26.5	-11.9	n.d.	n.d.
2016/8/28	VER16-03	2016St25GC1	51.3645	104.5459	729	60	Kedr-1	0.82	10.7	0.012	0.27	77	-75.4	-26.5	-12.4	-305.9	-195.5
2016/8/28	VER16-03	2016St25GC1	51.3645	104.5459	729	100	Kedr-1	2.10	31.8	0.014	0.36	66	-71.4	-26.5	-5.6	-301.0	-220.9
2016/8/28	VER16-03	2016St25GC1	51.3645	104.5459	729	140	Kedr-1	2.28	35.3	0.011	0.14	65	-70.2	-26.5	-9.1	-302.0	-215.7
2016/8/28	VER16-03	2016St25GC1	51.3645	104.5459	729	180	Kedr-1	3.02	49.0	0.009	0.23	62	-69.4	-26.5	-4.3	-304.3	-216.5
2016/8/28	VER16-03	2016St26GC1	51.3600	104.5304	810	30	Kedr-1	0.32	0.8	0.006	0.17	408	-88.8	-31.1	-11.4	n.d.	n.d.
2016/8/28	VER16-03	2016St26GC1	51.3600	104.5304	810	70	Kedr-1	0.97	4.2	0.008	0.43	231	-73.9	-31.8	-9.6	-304.1	n.d.
2016/8/28	VER16-03	2016St26GC1	51.3600	104.5304	810	110	Kedr-1	3.20	15.8	0.015	0.36	202	-69.1	-31.5	-4.9	-299.0	n.d.
2016/8/28	VER16-03	2016St27GC1	51.3600	104.5232	1020	30	Kedr-1	3.10	0.0	0.018	0.57	78585	-67.6	-35.5	-3.3	-323.6	n.d.
2016/8/28	VER16-03	2016St27GC1	51.3600	104.5232	1020	55	Kedr-1	9.42	0.1	0.051	1.71	81829	-69.3	n.d.	0.6	-311.3	n.d.
2017/8/25	VER17-03	2017St12GC1t	51.3625	104.5401	629	10	Kedr-1	0.28	7.2	0.005	0.34	39	-48.5	-26.0	-11.6	n.d.	-224.4
2017/8/25	VER17-03	2017St12GC1t	51.3625	104.5401	629	50	Kedr-1	13.16	671.1	0.527	1.40	20	-46.5	-26.4	-1.4	-275.9	-214.6
2017/8/25	VER17-03	2017St12GC1t	51.3625	104.5401	629	90	Kedr-1	7.11	222.5	0.109	1.25	32	-45.2	-26.1	1.0	-276.0	-214.1
2017/8/25	VER17-03	2017St12GC2	51.3621	104.5406	629	20	Kedr-1	11.07	183.3	0.145	0.63	60	-50.9	-27.1	1.8	-278.2	-214.0
2017/8/25	VER17-03	2017St12GC2	51.3621	104.5406	629	40	Kedr-1	3.85	96.4	0.075	0.82	40	-47.6	-27.0	10.4	-278.2	-209.9
2017/8/25	VER17-03	2017St12GC2	51.3621	104.5406	629	80	Kedr-1	1.93	51.7	0.032	1.37	37	-45.9	-27.0	14.5	-276.8	-208.0
2017/8/25	VER17-03	2017St12GC2	51.3621	104.5406	629	100	Kedr-1	3.28	82.3	0.037	2.27	40	-45.9	-27.0	15.5	-279.0	-207.6
2017/8/25	VER17-03	2017St12GC2	51.3621	104.5406	629	120	Kedr-1	4.41	119.3	0.051	1.38	37	-45.9	-27.3	13.3	-271.3	-206.2
2017/8/25	VER17-03	2017St12GC2	51.3621	104.5406	629	140	Kedr-1	2.37	72.1	0.042	1.34	33	-45.1	-27.4	12.9	-269.1	-208.8
2017/8/25	VER17-03	2017St12GC2	51.3621	104.5406	629	160	Kedr-1	3.53	319.3	0.250	1.29	11	-46.1	-26.8	14.6	-272.0	-210.7
2017/8/25	VER17-03	2017St12GC2	51.3621	104.5406	629	170	Kedr-1	1.77	114.8	0.071	0.91	15	-45.6	-27.4	13.4	-271.3	-210.6
2017/8/25	VER17-03	2017St12GC3	51.3630	104.5409	613	10	Kedr-1	0.01	1.6	0.004	0.14	5	-45.8	-29.5	-15.5	n.d.	n.d.
2017/8/25	VER17-03	2017St12GC3	51.3630	104.5409	613	50	Kedr-1	1.44	36.2	0.009	0.36	40	-54.2	-27.6	-13.2	-283.2	-205.7
2017/8/25	VER17-03	2017St12GC3	51.3630	104.5409	613	90	Kedr-1	6.63	178.6	0.019	0.51	37	-50.1	-27.6	-3.0	-278.6	-205.1
2017/8/25	VER17-03	2017St12GC3	51.3630	104.5409	613	130	Kedr-1	3.52	129.6	0.033	0.93	27	-46.5	-27.2	6.9	-276.6	-205.7
2017/8/25	VER17-03	2017St12GC3	51.3630	104.5409	613	170	Kedr-1	3.92	118.1	0.023	1.52	33	-46.4	-27.4	14.6	-272.1	-205.7
2017/8/25	VER17-03	2017St12GC3	51.3630	104.5409	613	210	Kedr-1	4.49	147.1	0.032	1.43	31	-45.5	-27.1	15.7	-276.1	-207.0
2017/8/25	VER17-03	2017St12GC3	51.3630	104.5409	613	230	Kedr-1	4.25	304.4	0.146	1.31	14	-45.6	-27.1	16.0	-274.0	-210.1
2017/8/25	VER17-03	2017St12GC3	51.3630	104.5409	613	250	Kedr-1	4.50	566.9	0.373	0.91	8	-45.7	-26.9	13.1	-274.6	-209.9

Table S2 (continue)

Date	Cruise Name	Core Name	Latitude [°N]	Longitude [°E]	Water Depth [m]	Sample Depth [cmblf]	Place	Molecular Composition					Isotopic Composition				
								CH_4 [mM/L]	C_2H_6 [uM/L]	C_3H_8 [uM/L]	CO_2 [mM/L]	$C_1/(C_2+C_3)$	CH_4 $\delta^{13}C$ [‰V-PDB]	C_2H_6 $\delta^{13}C$ [‰V-PDB]	CO_2 $\delta^{13}C$ [‰V-PDB]	CH_4 δ^2H [‰V-SMOW]	C_2H_6 δ^2H [‰V-SMOW]
2017/8/25	VER17-03	2017St12GC3	51.3630	104.5409	613	280	Kedr-1	3.87	152.5	0.048	1.33	25	-45.4	-27.1	16.7	-272.2	-208.5
2017/8/25	VER17-03	2017St12GC3	51.3630	104.5409	613	310	Kedr-1	3.23	149.0	0.077	2.05	22	-45.6	-27.6	22.5	-270.9	-210.0
2017/8/26	VER17-03	2017St12GC4t	51.3621	104.5402	631	10	Kedr-1	0.26	3.3	0.011	0.56	80	-55.6	-26.7	-11.7	n.d.	n.d.
2017/8/26	VER17-03	2017St12GC4t	51.3621	104.5402	631	50	Kedr-1	17.60	369.0	0.646	1.92	48	-54.6	-27.0	11.6	-287.6	-211.6
2017/8/26	VER17-03	2017St12GC4t	51.3621	104.5402	631	90	Kedr-1	11.46	276.3	0.444	1.66	41	-50.2	-26.6	11.9	-281.3	-209.7
2017/8/25	VER17-03	2017St13GC1	51.3550	104.5436	617	10	Kedr-2	0.01	0.0	0.004	0.14	225	-47.0	n.d.	-20.0	n.d.	n.d.
2017/8/25	VER17-03	2017St13GC1	51.3550	104.5436	617	50	Kedr-2	0.13	0.9	0.003	0.25	139	-68.7	-25.8	-16.7	n.d.	n.d.
2017/8/25	VER17-03	2017St13GC1	51.3550	104.5436	617	90	Kedr-2	1.08	11.8	0.006	0.33	91	-59.4	-26.9	-15.1	-284.9	-202.3
2017/8/25	VER17-03	2017St13GC1	51.3550	104.5436	617	130	Kedr-2	3.22	38.9	0.010	0.57	83	-57.0	-27.0	-9.3	-287.0	-209.5
2017/8/25	VER17-03	2017St13GC1	51.3550	104.5436	617	170	Kedr-2	5.03	66.0	0.015	0.85	76	-55.7	-27.2	-1.3	-288.8	-209.2
2017/8/25	VER17-03	2017St13GC1	51.3550	104.5436	617	210	Kedr-2	6.00	84.4	0.016	0.45	71	-54.9	-27.2	-2.9	-285.7	-207.3
2017/8/25	VER17-03	2017St13GC1	51.3550	104.5436	617	250	Kedr-2	6.40	95.7	0.017	0.41	67	-54.1	-27.1	1.7	-288.3	-206.5
2017/8/25	VER17-03	2017St13GC1	51.3550	104.5436	617	290	Kedr-2	5.98	94.7	0.016	0.21	63	-53.3	-27.1	1.4	-289.3	-206.5
2017/8/25	VER17-03	2017St13GC1	51.3550	104.5436	617	330	Kedr-2	6.76	112.0	0.020	0.48	60	-52.7	-27.1	6.4	-288.0	-205.7
2017/8/25	VER17-03	2017St13GC2	51.3540	104.5435	588	20	Kedr-2	0.00	0.1	0.003	0.14	52	-44.7	n.d.	-20.1	n.d.	n.d.
2017/8/25	VER17-03	2017St13GC2	51.3540	104.5435	588	60	Kedr-2	0.06	0.2	0.004	0.53	262	-57.2	-27.5	-14.7	n.d.	n.d.
2017/8/25	VER17-03	2017St13GC2	51.3540	104.5435	588	100	Kedr-2	1.75	24.5	0.011	0.73	71	-51.2	-26.7	-10.8	-275.0	-207.1
2017/8/25	VER17-03	2017St13GC2	51.3540	104.5435	588	140	Kedr-2	4.31	62.3	0.018	0.97	69	-48.2	-27.2	-5.0	-278.7	-206.1
2017/8/25	VER17-03	2017St13GC2	51.3540	104.5435	588	180	Kedr-2	4.95	87.3	0.015	0.72	57	-45.7	-27.1	5.6	-276.2	-208.1
2017/8/25	VER17-03	2017St13GC2	51.3540	104.5435	588	220	Kedr-2	11.37	238.0	0.039	2.49	48	-44.2	-26.7	18.3	-273.8	-209.8
2017/8/25	VER17-03	2017St13GC2	51.3540	104.5435	588	260	Kedr-2	6.53	155.3	0.029	2.17	42	-42.7	-26.5	22.7	-271.1	-205.5
2017/8/25	VER17-03	2017St13GC2	51.3540	104.5435	588	300	Kedr-2	3.80	105.1	0.020	2.07	36	-42.2	-26.7	24.2	-271.1	-204.5
2017/8/25	VER17-03	2017St13GC2	51.3540	104.5435	588	340	Kedr-2	3.30	95.1	0.019	1.70	35	-40.5	-26.3	23.8	-269.7	-201.9
2017/8/25	VER17-03	2017St13GC2	51.3540	104.5435	588	380	Kedr-2	4.24	114.6	0.026	2.08	37	-41.7	-26.7	26.3	-271.1	-205.8
2017/8/25	VER17-03	2017St13GC3	51.3536	104.5439	592	10	Kedr-2	0.81	12.5	0.007	0.16	65	-49.1	-27.6	-12.0	-268.7	-224.1
2017/8/25	VER17-03	2017St13GC3	51.3536	104.5439	592	50	Kedr-2	13.75	254.5	0.042	1.24	54	-44.9	-26.8	9.4	-272.1	-211.3
2017/8/25	VER17-03	2017St13GC3	51.3536	104.5439	592	90	Kedr-2	8.29	195.8	0.037	1.42	42	-42.8	-26.6	17.4	-269.2	-208.2
2017/8/25	VER17-03	2017St13GC3	51.3536	104.5439	592	130	Kedr-2	3.95	133.6	0.049	0.84	30	-42.1	-27.1	14.9	-270.0	-207.8
2017/8/25	VER17-03	2017St13GC3	51.3536	104.5439	592	165	Kedr-2	2.27	180.8	0.158	0.63	13	-41.7	-27.2	17.1	-269.3	-210.9
2017/8/25	VER17-03	2017St13GC3	51.3536	104.5439	592	200	Kedr-2	3.32	129.2	0.089	1.41	26	-41.8	-26.7	23.5	-271.1	-209.9
2017/8/25	VER17-03	2017St13GC3	51.3536	104.5439	592	210	Kedr-2	5.05	385.0	0.340	0.70	13	-42.0	-26.7	15.9	-273.8	-216.2
2017/8/25	VER17-03	2017St13GC3	51.3536	104.5439	592	240	Kedr-2	4.42	603.4	0.202	0.43	7	-43.2	-26.2	14.0	-278.3	-216.5
2017/8/25	VER17-03	2017St13GC4t	51.3517	104.5449	648	20	Kedr-2	0.08	0.2	0.006	0.20	428	-86.8	n.d.	-18.9	n.d.	n.d.
2017/8/25	VER17-03	2017St13GC4t	51.3517	104.5449	648	50	Kedr-2	0.29	1.8	0.005	0.36	160	-85.7	-28.1	-10.9	n.d.	n.d.
2017/8/25	VER17-03	2017St13GC4t	51.3517	104.5449	648	90	Kedr-2	0.79	6.1	0.010	0.99	129	-79.8	-27.8	-6.7	-318.5	n.d.
2017/8/25	VER17-03	2017St13GC4t	51.3517	104.5449	648	130	Kedr-2	1.42	12.4	0.011	0.45	114	-75.2	-27.8	-7.2	-301.0	-214.6
2017/8/25	VER17-03	2017St13GC4t	51.3517	104.5449	648	170	Kedr-2	1.57	14.7	0.012	0.22	107	-73.1	-27.8	-9.9	-295.7	-216.7
2017/8/25	VER17-03	2017St13GC4t	51.3517	104.5449	648	210	Kedr-2	2.81	27.4	0.017	0.22	103	-71.4	-27.7	-9.6	-300.4	-218.7
2017/8/25	VER17-03	2017St13GC5t	51.3533	104.5438	596	10	Kedr-2	0.44	8.0	0.012	0.31	55	-50.1	-27.8	-14.5	n.d.	n.d.
2017/8/25	VER17-03	2017St13GC5t	51.3533	104.5438	596	50	Kedr-2	8.53	219.4	0.029	0.74	39	-45.7	-27.7	-6.5	-274.9	-210.4
2017/8/25	VER17-03	2017St13GC5t	51.3533	104.5438	596	90	Kedr-2	11.41	311.5	0.034	0.92	37	-45.1	-27.2	3.8	-274.6	-211.0
2017/8/25	VER17-03	2017St13GC6t	51.3538	104.5440	596	20	Kedr-2	0.00	0.0	0.005	0.29	100	n.d.	n.d.	-15.4	n.d.	n.d.
2017/8/25	VER17-03	2017St13GC6t	51.3538	104.5440	596	50	Kedr-2	0.01	0.0	0.004	0.38	274	n.d.	n.d.	-13.7	n.d.	n.d.
2017/8/25	VER17-03	2017St13GC6t	51.3538	104.5440	596	90	Kedr-2	0.69	7.4	0.006	0.21	93	-49.6	-26.7	-16.0	n.d.	n.d.
2017/8/25	VER17-03	2017St13GC6t	51.3538	104.5440	596	130	Kedr-2	0.13	0.5	0.009	0.53	262	-55.8	-27.2	-14.8	n.d.	n.d.
2017/8/25	VER17-03	2017St13GC6t	51.3538	104.5440	596	170	Kedr-2	6.26	82.1	0.020	1.06	76	-47.3	-27.7	-5.8	-273.8	-208.0

Table S2 (continue)

Date	Cruise Name	Core Name	Latitude [°N]	Longitude [°E]	Water Depth [m]	Sample Depth [cmblf]	Place	Molecular Composition: CH_4 [mM/L]	C_2H_6 [uM/L]	C_3H_8 [uM/L]	CO_2 [mM/L]	$C_1/(C_2+C_3)$	Isotopic Composition: CH_4 $\delta^{13}C$ [‰V-PDB]	C_2H_6 $\delta^{13}C$ [‰V-PDB]	CO_2 $\delta^{13}C$ [‰V-PDB]	CH_4 $\delta^{2}H$ [‰V-SMOW]	C_2H_6 $\delta^{2}H$ [‰V-SMOW]
2017/8/25	VER17-03	2017St13GC6t	51.3538	104.5440	596	210	Kedr-2	16.59	266.2	0.043	1.55	62	-45.2	-27.7	5.6	-275.2	-206.3
2017/8/25	VER17-03	2017St13GC7t	51.3550	104.5437	617	10	Kedr-2	0.00	0.0	0.006	0.14	207	n.d.	n.d.	-19.1	n.d.	n.d.
2017/8/25	VER17-03	2017St13GC7t	51.3550	104.5437	617	50	Kedr-2	0.15	0.9	0.005	0.20	179	-64.7	-27.1	-16.9	n.d.	n.d.
2017/8/25	VER17-03	2017St13GC7t	51.3550	104.5437	617	90	Kedr-2	0.75	7.2	0.009	0.72	104	-57.5	-27.2	-10.3	n.d.	n.d.
2017/8/25	VER17-03	2017St13GC7t	51.3550	104.5437	617	130	Kedr-2	2.15	24.6	0.013	0.52	87	-55.4	-27.4	-9.3	-281.9	-210.8
2017/8/25	VER17-03	2017St13GC7t	51.3550	104.5437	617	170	Kedr-2	3.14	38.8	0.016	0.27	81	-54.6	-27.6	-7.4	-287.5	-209.5
2017/8/25	VER17-03	2017St13GC7t	51.3550	104.5437	617	210	Kedr-2	5.93	80.1	0.023	0.45	74	-54.6	-27.7	-1.8	-290.1	-210.8
2017/8/25	VER17-03	2017St13GC7t	51.3550	104.5437	617	245	Kedr-2	9.65	134.9	0.033	0.43	71	-53.4	-27.8	0.0	-290.0	-207.0

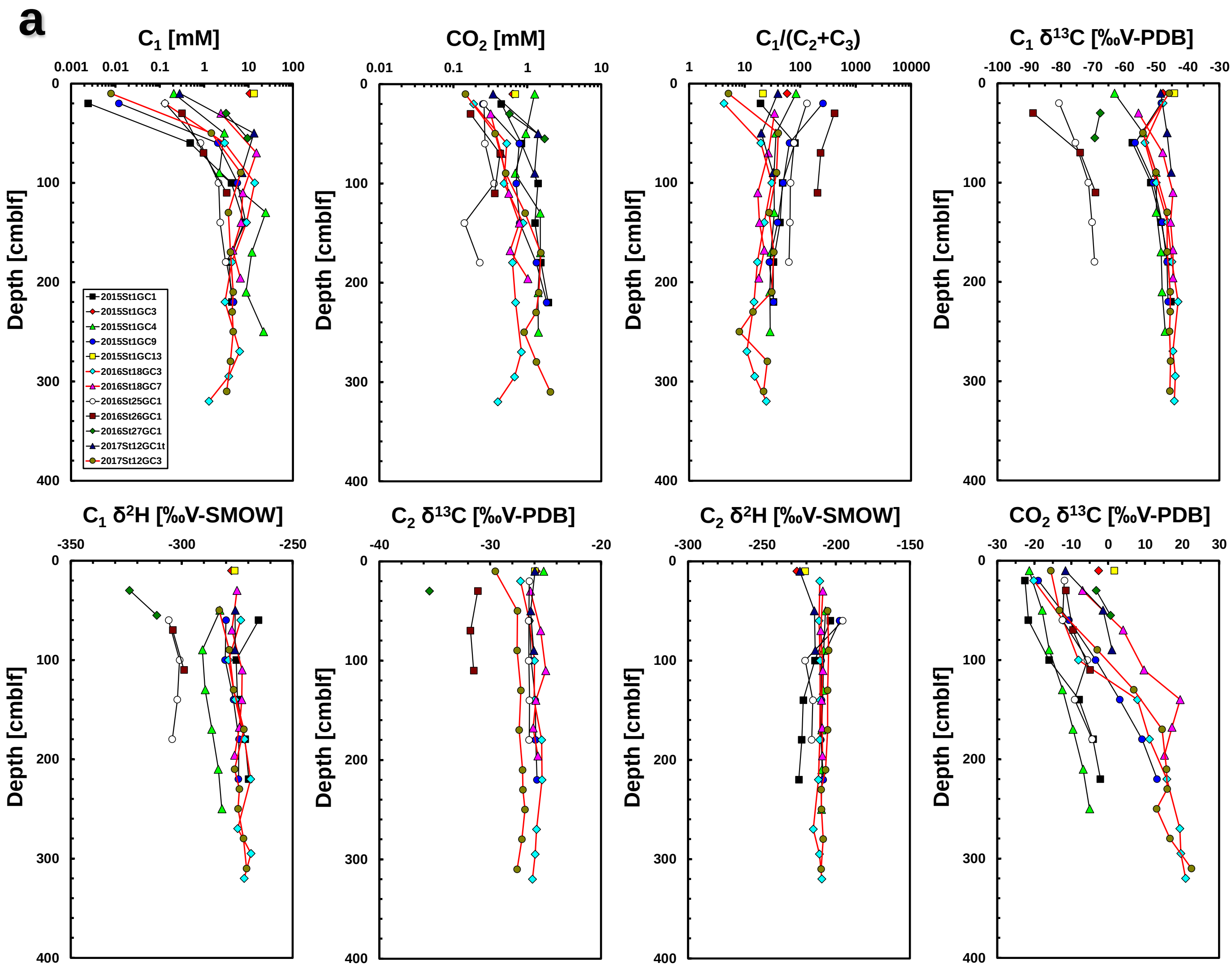

Figure S1. Depth profiles of C_1 and CO_2 concentrations, C_1 / (C_2 + C_3) values, C_1 $\delta^{13}C$, C_1 δ^2H, C_2 $\delta^{13}C$, C_2 δ^2H and CO_2 $\delta^{13}C$ in the headspace gas. **a** northern area of Kedr-1 and its periphery, **b** southern area of Kedr-1 (mainly gas hydrate cores), **c** Kedr-2 area and its periphery. cmblf, centimetres below lake floor.

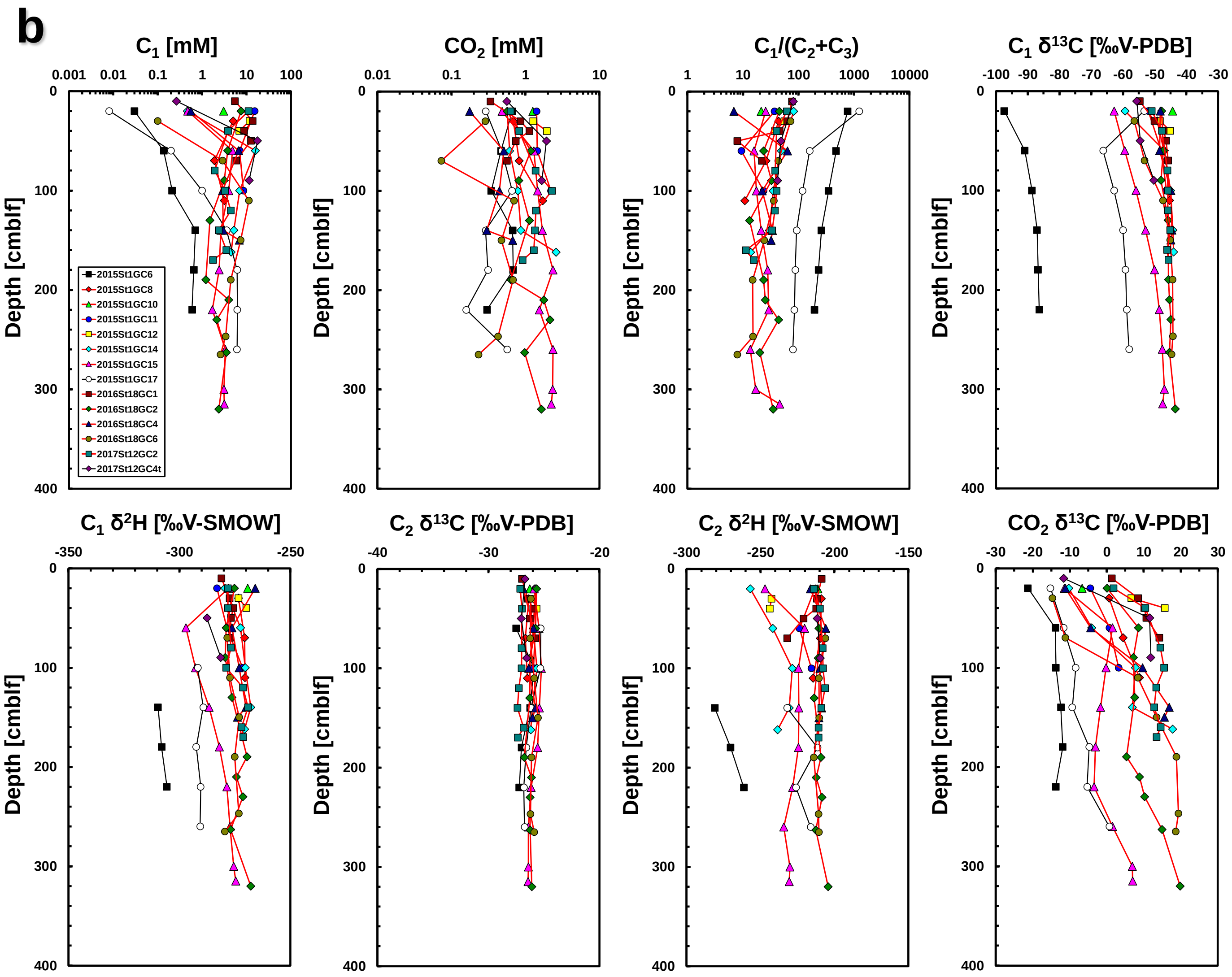

Fig. S1 (continue)

c

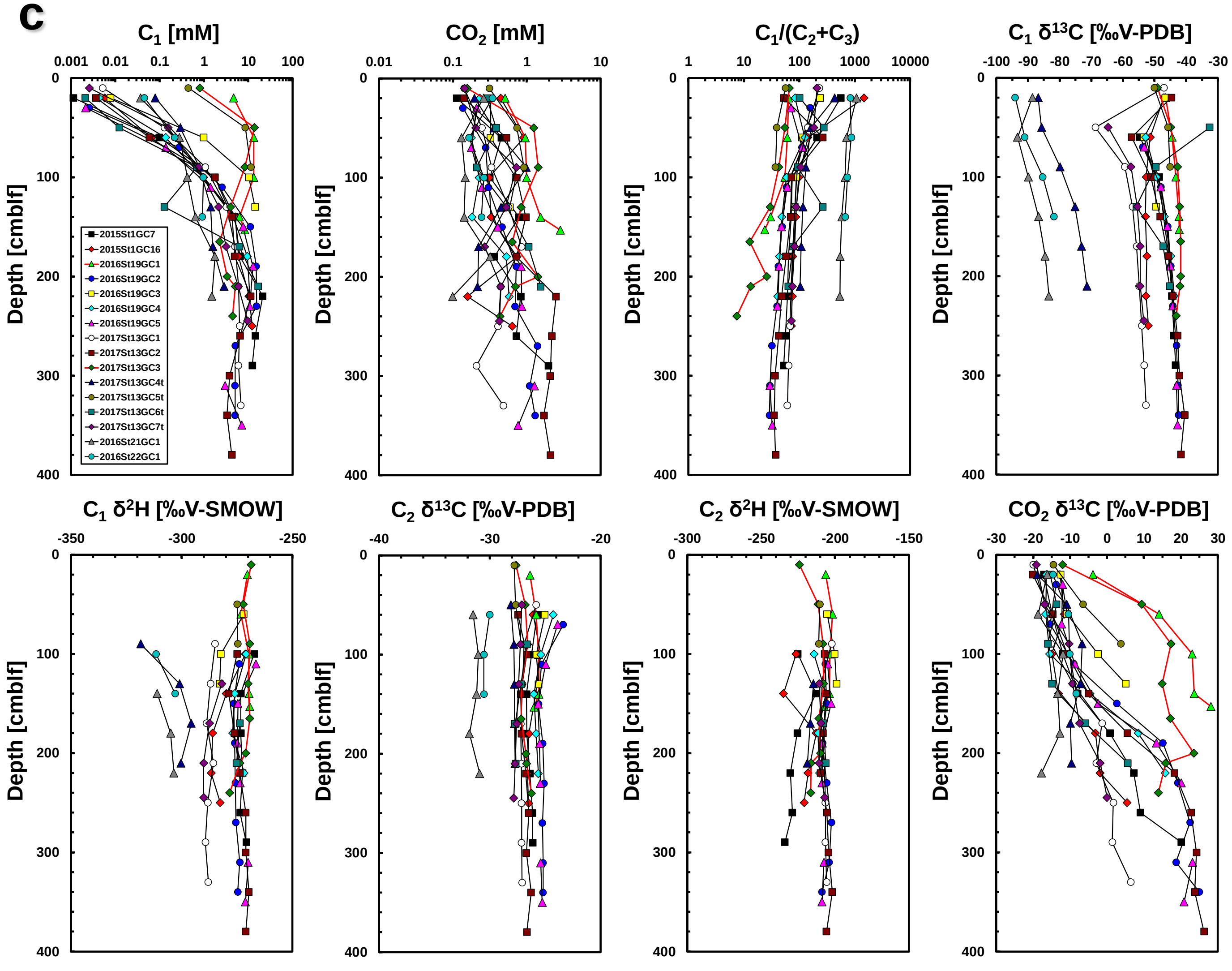

Fig. S1 (continue)
